# A comparison of Illumina and Ion Torrent sequencing platforms in the context of differential gene expression

**DOI:** 10.1186/s12864-017-4011-0

**Published:** 2017-08-10

**Authors:** Nicholas F. Lahens, Emanuela Ricciotti, Olga Smirnova, Erik Toorens, Eun Ji Kim, Giacomo Baruzzo, Katharina E. Hayer, Tapan Ganguly, Jonathan Schug, Gregory R. Grant

**Affiliations:** 1 Institute for Translational Medicine and Therapeutics, Perelman School of Medicine, University of Pennsylvania, Philadelphia, PA 19104 USA; 2 Department of Systems Pharmacology and Translational Therapeutics, Perelman School of Medicine, University of Pennsylvania, Philadelphia, PA 19104 USA; 3 Institute for Diabetes, Obesity and Metabolism, Perelman School of Medicine, University of Pennsylvania, Philadelphia, PA 19104 USA; 4 Department of Genetics, Perelman School of Medicine, University of Pennsylvania, Philadelphia, PA 19104 USA; 5 Penn Genomic Analysis Core, Perelman School of Medicine, University of Pennsylvania, Philadelphia, PA 19104 USA; 6 Department of Information Engineering, University of Padova, Padova, Italy; 7 Department of Biomedical and Health Informatics, The Children’s Hospital of Philadelphia, Philadelphia, PA 19104 USA; 8 Abramson Cancer Center, Perelman School of Medicine, University of Pennsylvania, Philadelphia, PA 19104 USA

**Keywords:** RNA-Seq, Ion Torrent, Illumina, Differential expression, Pathway analysis

## Abstract

**Background:**

Though Illumina has largely dominated the RNA-Seq field, the simultaneous availability of Ion Torrent has left scientists wondering which platform is most effective for differential gene expression (DGE) analysis. Previous investigations of this question have typically used reference samples derived from cell lines and brain tissue, and do not involve biological variability. While these comparisons might inform studies of tissue-specific expression, marked by large-scale transcriptional differences, this is not the common use case.

**Results:**

Here we employ a standard treatment/control experimental design, which enables us to evaluate these platforms in the context of the expression differences common in differential gene expression experiments. Specifically, we assessed the hepatic inflammatory response of mice by assaying liver RNA from control and IL-1β treated animals with both the Illumina HiSeq and the Ion Torrent Proton sequencing platforms. We found the greatest difference between the platforms at the level of read alignment, a moderate level of concordance at the level of DGE analysis, and nearly identical results at the level of differentially affected pathways. Interestingly, we also observed a strong interaction between sequencing platform and choice of aligner. By aligning both real and simulated Illumina and Ion Torrent data with the twelve most commonly-cited aligners in the literature, we observed that different aligner and platform combinations were better suited to probing different genomic features; for example, disentangling the source of expression in gene-pseudogene pairs.

**Conclusions:**

Taken together, our results indicate that while Illumina and Ion Torrent have similar capacities to detect changes in biology from a treatment/control experiment, these platforms may be tailored to interrogate different transcriptional phenomena through careful selection of alignment software.

**Electronic supplementary material:**

The online version of this article (doi:10.1186/s12864-017-4011-0) contains supplementary material, which is available to authorized users.

## Background

RNA-Sequencing (RNA-Seq) broadly refers to a family of experimental techniques that give researchers the ability to study the transcriptional landscapes of cells and tissues quantitatively by exploiting high throughput sequencing technology. Currently, the most commonly used sequencing platforms are provided by Illumina, which uses a fluorescence-based paradigm for reading the bases in a nucleotide sequence. One alternative option is provided by Ion Torrent, which is built around the use of pH measurements to read nucleotide sequences. In addition to the distinct sequencing technologies used by these two platforms, there are smaller differences in the types of data they generate. In Illumina data all sequence reads generated during a single experiment have the same lengths, while the lengths of Ion Torrent reads vary. Additionally, the current generation of Illumina instruments can generate sequence reads from both ends of a fragment (“paired-end” reads), while Ion Torrent cannot.

Prior studies have compared these two sequencing platforms for various applications including genome sequencing, RNA-Seq, and microbiome profiling [1–5]. These studies provide a basic indication of the error rates and reproducibility these platforms achieve, however there are two motivations for performing the present analysis.

First, in the intervening years both Illumina and Ion Torrent have released platform updates in the form of improved sequencing chemistry, nucleotide detection, and throughput. With the rapid pace of development in these sequencing technologies, we must continue to compare these two platforms in order to maintain an accurate understanding of their relative performances. This continued re-assessment is of particular importance as researchers adapt both of these sequencing platforms for use in the clinical setting [6, 7].

Second, the previous studies that focused on comparing the Illumina and Ion Torrent platforms for the purposes of RNA-Seq expression analysis used the Universal Human Reference RNA (UHRR) as source RNA [2, 8, 9]. The UHRR is a mixture of ten human cell lines derived from various tissues [10] and has served as a standard for many studies benchmarking microarray and RNA-Seq performance [11, 12]. While these well-studied reference samples have proven useful for reproducibility assessments, they are derived from immortalized cell lines and are therefore somewhat removed from tissue-derived RNA samples collected in vivo. Additionally, many of these benchmarking studies combine different reference RNA mixtures at various quantities to assess differential expression. Again, while it has its uses, comparing these different UHRR mixtures is closer to performing a between-tissue comparison, in which we expect to see gross transcriptional changes between samples. Lastly, multiple UHRR samples are effectively technical replicates of one another, and therefore lack the biological variability that will affect the results of most RNA-Seq experiments in practice.

Arguably, one of the most common designs for RNA expression profiling experiments involves a single tissue or cell type, and varying genotypes or treatments. Generally, the transcriptional differences we expect to see between “treatment” and “control” conditions are more subtle than those we see between universal reference samples. Therefore, in order to evaluate the two platforms in the context of this end point, we used the Illumina and Ion Torrent sequencing platforms to assess the effects of IL-1β treatment on the mouse liver transcriptome. We evaluated these platforms at three levels of complexity: 1) individual read alignments and expression quantification, 2) differential expression detection, and 3) pathway-level analysis. Here we seek to determine if/how choosing between the Illumina and Ion Torrent sequencing platforms will affect the biological conclusions a researcher derives from the data, for which concordance at the pathway level is most relevant.

## Results

### A treatment/control experimental design to compare platforms

**Fig. 1:**
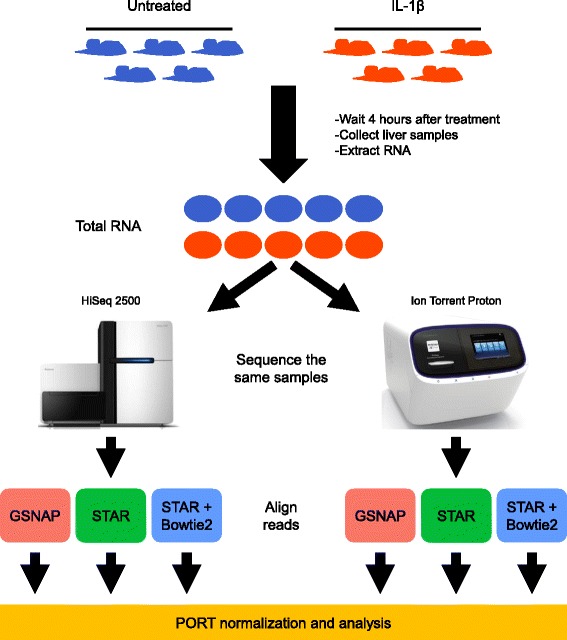
A treatment/control experimental design. Ten mice were treated with IL-1β (*n* = 5), or saline (*n* = 5; referred to as untreated). Four hours after treatment, the mice were sacrificed, liver samples were collected, and total RNA was extracted from the tissue. At this point, aliquots of the same RNA sample were sequenced on both an Illumina HiSeq 2500 and an Ion Torrent Proton. Next, RNA-Seq reads from each platform were aligned using three alignment algorithms: 1) GSNAP, 2) STAR, and 3) STAR, followed by Bowtie2 to align reads not mapped by STAR (STAR + Bowtie2). Lastly, all aligned data were normalized using the **P**ipeline **O**f **R**NA-Seq **T**ransformations (PORT)

We sought to compare the Illumina and Ion Torrent sequencing platforms using a treatment/control experimental paradigm (see *Methods* section for details). Briefly, we treated ten male mice with either 20 μg/Kg of IL-1β (*n* = 5) or saline (*n* = 5; hereafter referred to as untreated), and then collected liver samples from these mice four hours after treatment (Fig. 1). After extracting RNA from these liver samples, we prepared platform specific libraries from all ten RNA samples and sequenced them using both an Illumina HiSeq 2500 and an Ion Torrent Proton. We aligned the raw sequencing data for both platforms using both GSNAP [13] and STAR [14] (see Additional file 1: (Supplementary Methods) for alignment parameters). These algorithms showed the best performance in a recent benchmarking analysis of RNA-Seq alignment algorithms performed by our lab [15]. We also performed a sequential analysis aligning reads with STAR first, and then used Bowtie2 [16] to align any reads not mapped by STAR, since previous work found this strategy performed well for Ion Torrent RNA-Seq data [17, 18]. Next, we used the **P**ipeline **O**f **R**NA-Seq **T**ransformations (PORT) [19] to both normalize and quantify the aligned RNA-Seq reads separately for each combination of sequencing platform and alignment algorithm.

All aligners achieved high percentages of uniquely mapped reads in both Illumina and Ion Torrent data (Additional file 2: Figure S1; Additional file 3: Table S1), with the majority of reads mapping to exonic regions. The STAR alignments showed the smallest percentage of uniquely aligned reads in both platforms, while GSNAP and the combination of STAR + Bowtie2 tended to show the largest. The lower performance of STAR in the Ion Torrent data may be due to the variable read lengths present in the Ion Torrent data (Additional file 4: Figure S2; Additional file 5: Table S2). The STAR parameter “sjdbOverhang” is used when creating the STAR genome indexes and its value is generally determined by the read length of the dataset. It is possible that further tweaking of this parameter may improve the performance of STAR on Ion Torrent data. Here we used the same genome indexes for both the Illumina and Ion Torrent data to facilitate direct comparisons between the two platforms. Despite these differences we continued to use data from all alignment schemes in our analyses, for comparison purposes.

### Depth of coverage comparison

**Fig. 2:**
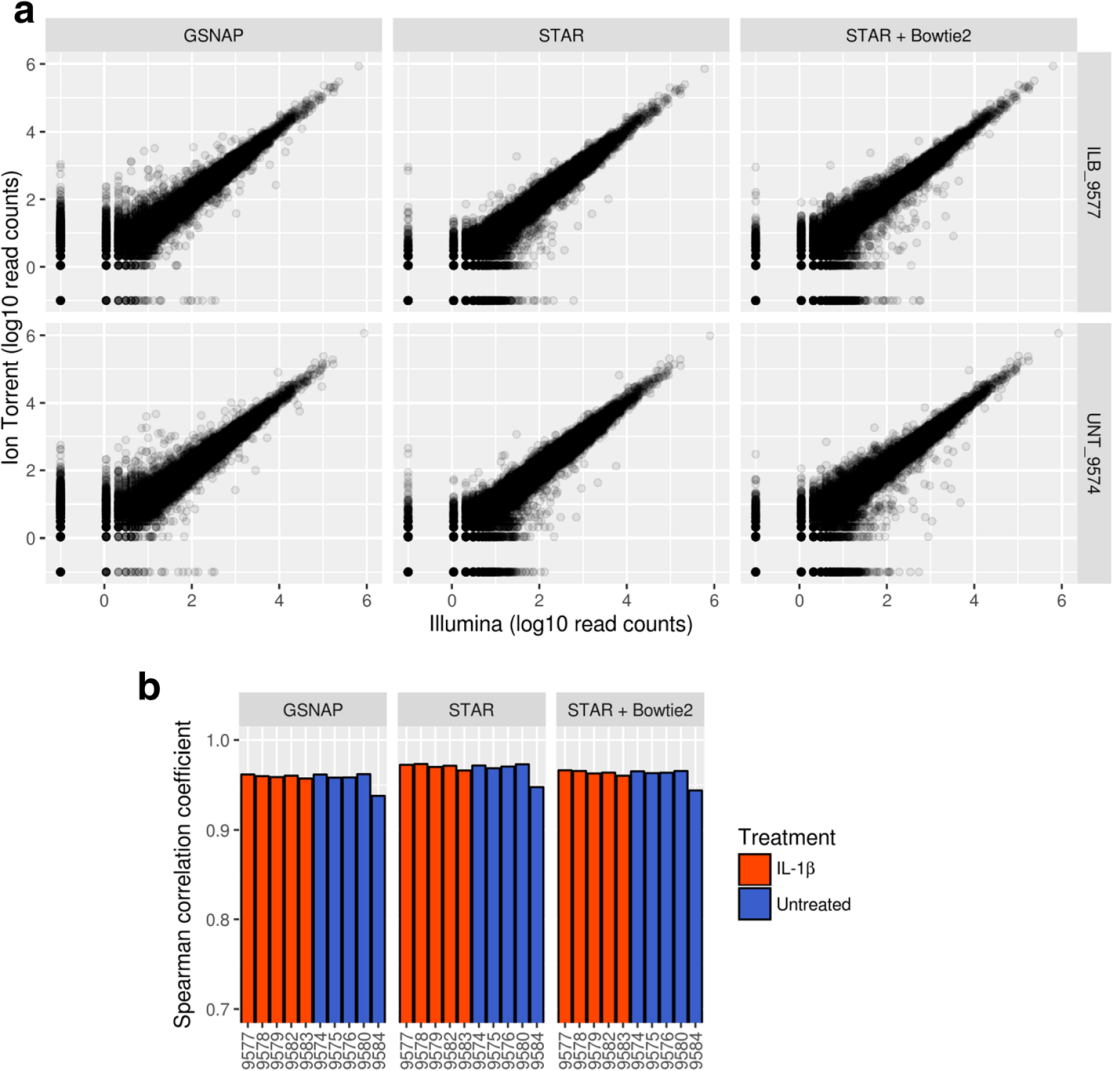
Read count comparison between platforms**. a** Scatterplots comparing the gene-level read counts between Illumina (x-axis) and Ion Torrent (y-axis). Results are displayed for two representative samples (IL-1β treated 9577 and untreated 9574), across all three alignment algorithms. Both axes are scaled to log10 of the PORT-normalized read counts. **b** Spearman correlation coefficients comparing Illumina and Ion Torrent gene-level read counts. Correlation coefficients are displayed for all samples and colored according to treatment group (IL-1β = orange; untreated = blue). The one sample showing a slightly reduced correlation is untreated 9584

We begin the platform comparison at the levels of read alignment and gene quantification. For all analyses, we limit our results to a set of “detected” genes (see *Methods* section for full details). Briefly, we define a gene as “detected” if it is mapped by at least five reads in five of the samples, in any combination of platform and alignment algorithm. After this filtering step, we directly compared gene-level read counts from the Illumina and Ion Torrent data alignments. For any given sample, we see a strong linear relationship between the read counts from both platforms (Fig. 2a - representative samples; Additional file 6: Figure S3 - all samples). Additionally, for each RNA sample the Spearman correlation between the read counts of each platform ranged from 0.9380 to 0.9737 (Fig. 2b; Additional file 7: Table S3), underscoring the strong agreement between the platforms at this level. While one sample (untreated 9584) consistently had lower Spearman correlations than the others, it still showed a strong correlation (~0.93–0.94). Interestingly, this same sample also displayed a shifted Ion Torrent read-length distribution relative to the other samples (Additional file 4: Figure S2), which indicates that differences in read length between libraries in Ion Torrent could be a source of technical variability. That being said, the high correlation coefficients we saw across all samples are in agreement with previous work comparing these two platforms [8]. We observed this agreement consistently across all alignment algorithms.

In addition to the gene-level agreement between the Illumina and Ion Torrent platforms, they also showed similarity with respect to reads mapping to ribosomal RNA (rRNA)—commonly present even after poly-A selection—and the mitochondrial chromosome (chrM). Both platforms identified a down-regulation of reads mapping to chrM in the mice treated with IL-1β (Additional file 8: Figure S4, top), although the effect was more subtle in the Ion Torrent data. This falls in line with previous findings that IL-1β affects mitochondrial function in the liver, inhibiting hepatic ATP production [20]. Neither platform showed a treatment-specific effect in the number of rRNA reads (Additional file 8: Figure S4, bottom), though Ion Torrent tended to have a smaller percentage of rRNA-mapping reads than Illumina. Again, these patterns are present in data mapped by each alignment algorithm. Taken together, these results suggest the two sequencing platforms agree substantially at the level of alignments and gene quantification.

### Differential expression comparison agrees across platforms

To identify genes that are differentially expressed between our two treatment conditions we used a two-sided Mann-Whitney *U* test [21], followed by a Benjamini-Hochberg correction for multiple testing [22]. We chose this classical method for three primary reasons: 1) This is a widely used and relatively uncontroversial approach, 2) with five replicates in each condition we had enough samples to generate significant *p*-values from the permutation procedure used by the Mann-Whitney *U* test, and 3) many of the modern methods for identifying differentially expressed genes (DEGs) are built on top of assumptions and models derived largely from Illumina data. This is not to say that these methods are invalid for use with Ion Torrent data, but we wanted to avoid using methods that might make assumptions specific to one of the sequencing platforms.

**Fig. 3:**
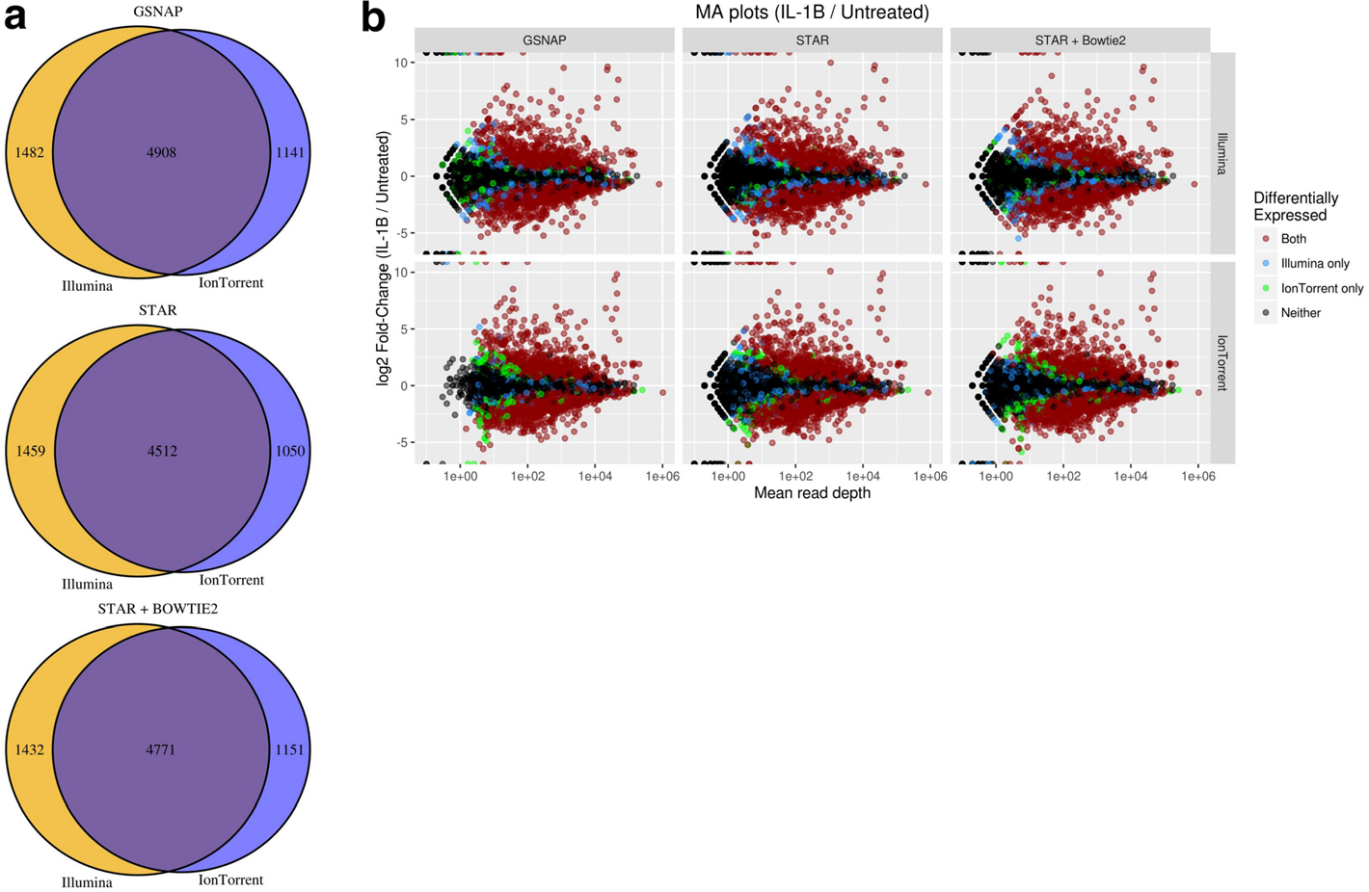
Differential expression comparison agrees across platforms. Within each combination of platform and aligner, differentially-expressed genes (DEGs) were identified using a two-sided Mann-Whitney test, followed by a Benjamini-Hochberg (BH) correction for multiple testing. Genes with BH *q*-values <0.05 were identified as differentially expressed. **a** The overlap in DEGs between Illumina and Ion Torrent for each aligner. **b** MA plots for every combination of platform and aligner. Within each aligner, genes are colored according to the platform in which they were identified as DEGs

We define a gene as significant if it has a *q*-value ≤0.05 (i.e. a Benjamini-Hochberg false discovery rate no more than 5%). All combinations of alignment algorithm and platform discovered roughly 5500–6400 differentially expressed genes, with the Illumina data detecting 280–400 more DEGs than the Ion Torrent data (Fig. 3a; Additional file 9: Table S4). Within a given alignment algorithm both platforms had ~76–81% of their DEG lists in common, indicating a moderate concordance. Focusing on those genes detected as differentially expressed by only one of the platforms, we found the majority were at the fringes of detectability owing to their low expression levels or small fold-changes (Fig. 3b - blue and green dots). Thus the typical gene not in the intersection was just below our significance cutoff in one platform. We hypothesize that the majority of these platform-specific DEGs that are truly differential would likely be detected by both platforms with additional sequencing depth. To test this hypothesis, we randomly down-sampled our normalized GSNAP data from both platforms to various levels, repeated the Mann-Whitney DE analysis in each down-sampled dataset, and compared the agreement between the two platforms as a function of coverage depth (see Additional file 1: Supplementary Methods for full details). Our down-sampling experiment showed that as read depth increases, so does the percentage of total DEGs identified by both platforms (Additional file 10: Figure S5), which provides initial evidence in support of this hypothesis. While these increases in concordance may seem modest, this down-sampling experiment uses read depths at the lower end of the spectrum (6–12 million reads; 2-fold change in read depth) for most RNA-seq experiments. We also repeated our DE analysis using the *limma* package [23] to assess how an algorithm specifically designed for expression data would perform in these two platforms. We found *limma* identified nearly all of the DEGS from our Mann-Whitney analyses (Additional file 9 Table S4; Additional file 11: Figure S6 ), in addition to identifying platform-specific DEGs not originally found by Mann-Whitney. These differences are likely due to the differing statistical power of the tests underlying these two methods. Interestingly, many DEGs identified as platform-specific using Mann-Whitney were identified in both platforms using *limma*. This also supports our hypothesis that many of these platform-specific DEGs are the result of sequencing depth and experimental/biological noise, and would otherwise be detectable in both platforms. We continue to use the Mann-Whitney DE results for the remainder of this manuscript, for the reasons we outlined above (it is agnostic to platform and alignment method).

The DEGs identified by both platforms were among those with the highest expression levels and largest fold-change values (Fig. 3B - red dots). Comparing the fold-change values for the DEGs between platforms directly, we again found good agreement for those DEGs identified in both Illumina and Ion Torrent (Additional file 12: Figure S7; Additional file 13: Table S5). The fold-change values for platform-specific DEGs tended to be larger for the platform in which they were detected, though they still showed strong, positive correlations between the two platforms. Thus, both platforms are equally capable of identifying the most significant differences in gene expression and are in good agreement at the level of DEG detection.

### Both platforms are in agreement about the top enriched pathways

**Fig. 4:**
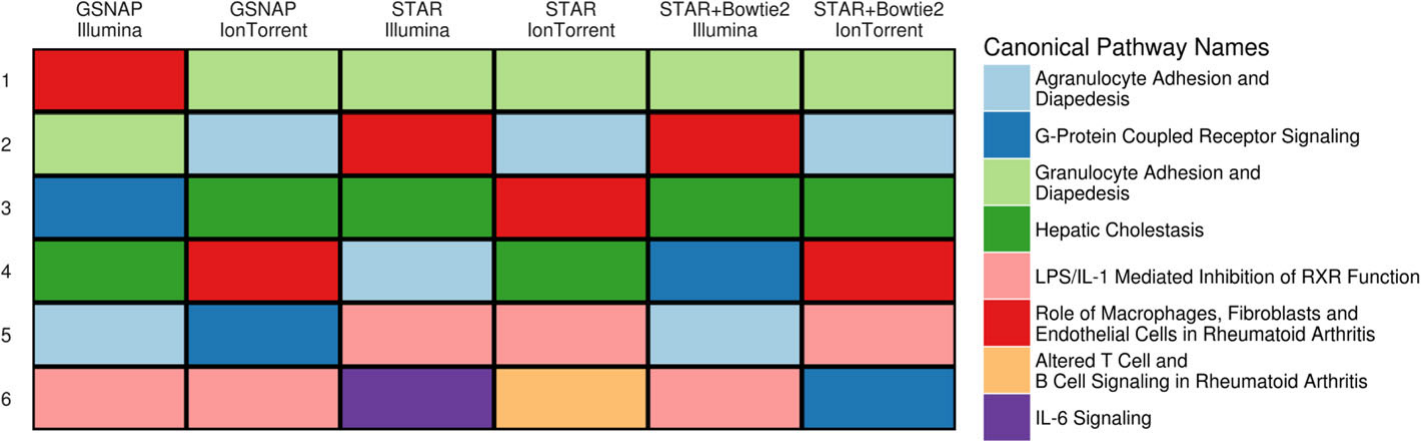
Both platforms show good agreement among the top enriched pathways. Ingenuity Pathway Analysis was performed separately on the lists of DEGs identified by each combination of aligner and platform. This figure presents the top 6 canonical pathways with significant enrichment in each dataset (ordered by enrichment *p*-value)

To investigate how well the results from both platforms agree at the level of biological systems, we used Ingenuity Pathway Analysis (IPA) [24], which identifies the pathways and biological systems most affected by the IL-1β treatment. The IPA tool uses a curated database of literature and experimental results to identify the pathways and biological functions enriched among a list of DEGs. We collected lists of DEGs from every combination of platform and alignment algorithm and analyzed each list separately using IPA. Both platforms showed strong enrichment of pathways related to the inflammatory response, regardless of alignment algorithm (Fig. 4; Additional file 14: Table S6). These top pathways include *granulocyte adhesion and diapedesis*, *hepatic cholestasis*, as well as various interleukin signaling pathways. Given the proinflammatory role of IL-1β, this is in line with our expectations [25–28]. Perhaps most importantly, the IPA results across all datasets identify IL-1β as one of the top two upstream regulators (Additional file 15: Table S7). In summary, given the strong agreement among enriched biological pathways between both platforms, a scientist using either sequencing technology would ultimately reach the same systems-level conclusions about the effects of IL-1β on liver function.

### Platform and aligner choice affects detection of a subset of genes

**Fig. 5:**
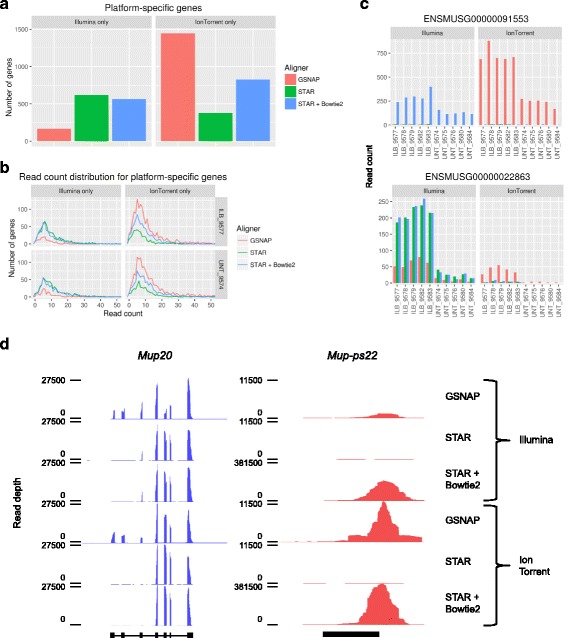
Differential gene detection due to platform/aligner choice. **a** Bar graphs displaying the number of genes detected exclusively by Illumina or Ion Torrent. These numbers are displayed for all three alignment algorithms. Detected genes are defined as those with at least 5 reads in 5 of the samples. **b** Distribution of read counts for platform-specific genes are displayed for two representative samples (IL-1β treated 9577 and untreated 9574), across all three alignment algorithms. The majority of platform-specific genes have less than 50 reads, so the graph’s x-axis is limited to the [0,50] range for display purposes. **c** Expression traces for two representative genes showing differential detection between platforms/aligners. Expression plots are colored according to aligner. **d** Coverage plots for a gene/pseudogene pair with significant differenc﻿es across aligner/platform; *Mup20* (left; blue) and *Mup-ps22* (right; red) from a representative sample (untreated 9574) across all combinations of platform and aligner. Gene models for *Mup20* and *Mup-ps22* are displayed in the sense orientation (5′ → 3′) below the coverage plots. Note, the loci displayed for *Mup20* and *Mup-ps22* are 22,000 bp and 2000 bp in length, respectively

While the majority of our analyses indicate a strong agreement between both platforms, we did observe that some genes are detected in a platform-specific manner. Examining the data from each of the alignment algorithms separately, we found the STAR alignments yielded the most platform-specific genes in the Illumina data, while GSNAP yielded the most platform-specific genes in the Ion Torrent data (Fig. 5a; Additional file 16: Table S8). Additionally, the bulk of these platform-specific genes are mapped by less than 10 reads (Fig. 5b - representative samples; Additional file 17: Figure S8 - all samples). This suggests that the genes which are detected in a platform-specific manner are expressed at low levels. We hypothesize that increasing read depth or performing a replicate of this experiment would allow for detection of these genes in both platforms.

Looking specifically at the DEGs, we compared the length, number of exons, average exon length, and GC content of genes identified by both platforms, Illumina only, Ion Torrent only, and neither platform. While there were no differences in the majority of these metrics between these groups of DEGs, we did observe a trend in the GC content (Additional file 18: Figure S9). The DEGs detected in both platforms had a GC content centered on 50%, while the Illumina- and Ion Torrent-specific DEGs tended to have lower and higher GC contents, respectively. Both platforms have known GC biases [1, 29, 30] which could be contributing to these platform-specific differences.

In addition to read counts, we also compared the biotypes of the various genes detected in these data (Additional file 19: Table S9). While over 90% of genes detected by both platforms were protein-coding, up to 50% of the platform-specific genes were classified as pseudogenes. Among these platform-specific genes, the exact percentage of protein coding and pseudogenes varies substantially depending upon both the sequencing platform and the aligner. This observation hints at an interaction between platform and aligner that impacts a researcher’s ability to detect particular genes.

There are a few platform-specific genes that exhibited higher depth of coverage (> 100 mapped reads; Additional file 16: Table S8). However, many of these genes while platform-specific in data generated from one aligner were detected by both platforms when considering all of the aligners together (Fig. 5c - representative examples). Curiously, in many of these cases the choice of alignment algorithm had substantial effects on the depth of coverage for these platform-specific genes. Consider two representative examples of differentially expressed genes: *Serpina3e-ps* and *Btg3*. We detected *Serpina3e-ps* (Fig. 5c - top) with both platforms using STAR + Bowtie2, with neither platform using STAR, and only with the Ion Torrent data using GSNAP. Similarly, we detected *Btg3* (Fig. 5c - bottom) with both platforms using GSNAP and STAR + Bowtie2, but with Illumina only when using STAR. In both of these cases, our ability to detect these genes was dependent both on our choice of sequencing platform, as well as our choice of alignment algorithm.

### An interaction between platform and aligner affects detection of a gene/pseudogene pair

One particularly startling example of this platform/aligner interaction is the gene/pseudogene pair of *Mup20* and *Mup-ps22*. We detected *Mup20* at very high expression levels using all combinations of platform and aligner (Fig. 5d - left, a representative sample). This is expected, as the major urinary protein (MUP) genes are expressed at very high levels in the livers of male mice [31]. Looking more closely at the coverage plots (displaying read depth across the length of each gene locus, rather than the total number of reads mapped to each gene), we did see that detection of the 5′ exons is affected by aligner choice, with the GSNAP-aligned data yielding the highest 5′ coverage. The related pseudogene *Mup-ps22* was detected at very low levels with STAR, substantially higher levels with GSNAP, and at extremely high levels with STAR + Bowtie2 (Fig. 5d - right). Furthermore, even looking within the GSNAP- and STAR + Bowtie2-aligned data, *Mup-ps22* was detected at a substantially higher level in the Ion Torrent data than in the Illumina data. We have confirmed the expression of both genes at detectable levels by qPCR (Additional file 20: Figure S10). This is a particularly extreme example of the phenomenon we observed previously, where our selection of both alignment algorithm and platform determines whether a gene is detected.

**Fig. 6:**
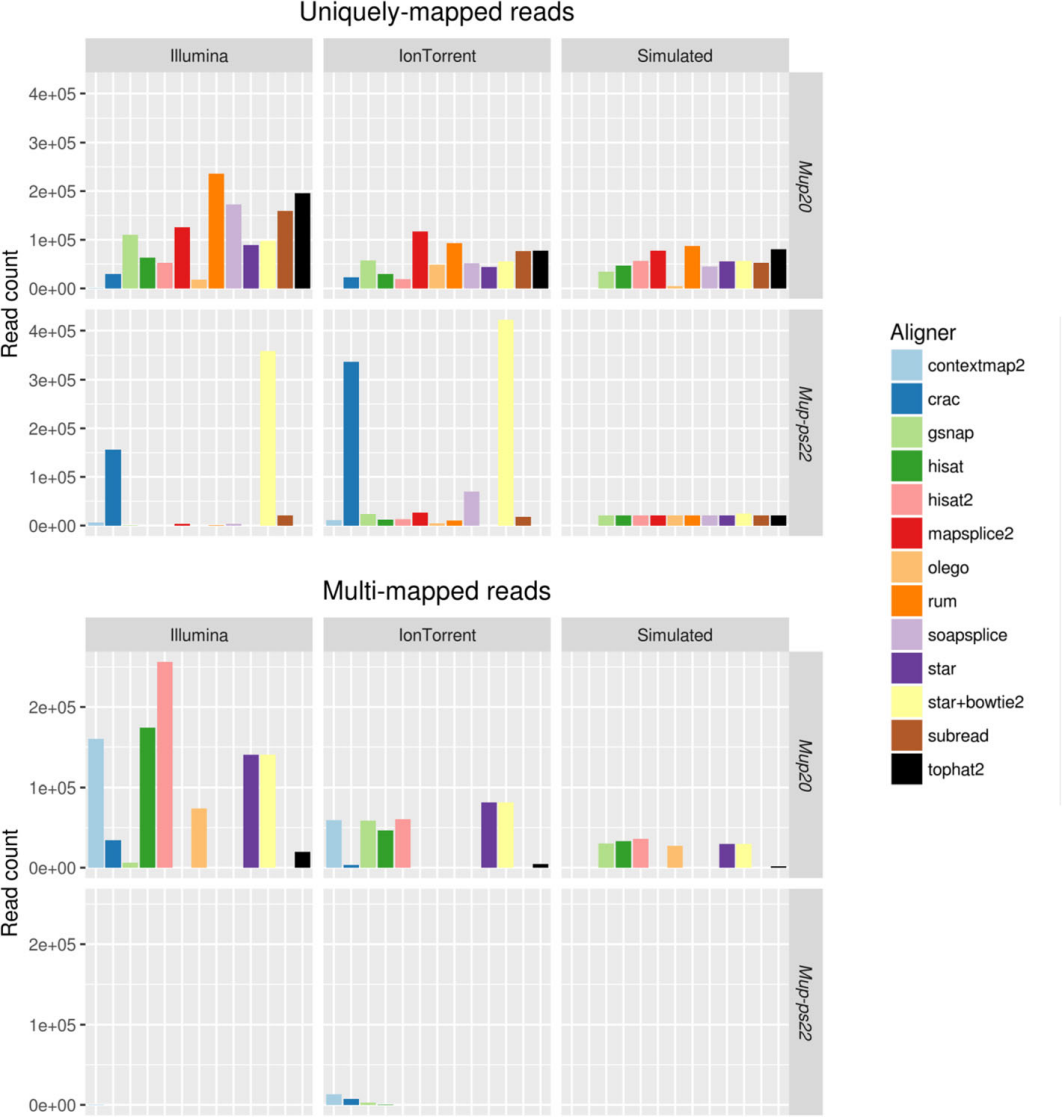
Using simulated data to examine platform/aligner interaction. For each sequencing platform, all reads aligned by GSNAP, or STAR + Bowtie2 to *Mup20* or *Mup-ps22* from sample 9574 (untreated) were extracted. These data were re-aligned using STAR + Bowtie2 and the twelve most popular aligners, according to a survey of the literature. Additionally, simulated RNA-Seq reads were generated from both of these genes. *Mup20* expression was simulated at three times the level of *Mup-ps22*. This figure displays the number of uniquely-mapped (top) and multimapped (bottom) reads aligned to *Mup20* or *Mup-ps22*

To further examine this platform/aligner interaction, we extracted all reads from untreated sample 9574 aligned by GSNAP, or STAR + Bowtie2 to either *Mup20* or *Mup-ps22*. Next, we used the STAR + Bowtie2 combination and the twelve most popular alignment algorithms, as determined by a recent survey of the literature [15], to map these reads to the reference genome. Interestingly, we found drastically different levels of expression for both genes across all alignment algorithms (Fig. 6). It is possible this variability in coverage could be explained by the differing strategies the aligners use to declare read alignments as ambiguous. However, the majority of aligners assigned few, if any, multimappers to *Mup-ps22* in either platform. It is also possible that we introduced a bias by using the reads aligned by GSNAP and STAR + Bowtie2 as input for the other alignment algorithms. To test for this effect we aligned all reads from sample 9574 using all thirteen aligners. We found that while the overall read depth at these loci is increased when using all of the reads, there was no change in the platform/aligner effect we observed above (Additional file 21: Figure S11). Taken together these observations provide further evidence that the choice of platform and aligner can affect our ability to resolve expression originating from different genomic loci. Furthermore, these differences are not due solely to an aligner’s ability to resolve multimapped reads.

To further investigate the differential behavior of the aligners, we generated simulated RNA-Seq reads from each of these two genes using the Benchmarker for Evaluating the Effectiveness of RNA-Seq Software [32]. We simulated ~100,000 reads, with *Mup20* expressed at three times the level of *Mup-ps22*, and aligned the resulting reads using the same thirteen alignment algorithms as with our real data (Fig. 6 - right column). Again we observed differences between the alignment algorithms in their ability to map reads to each of these two genes. Furthermore, the majority of aligners, including the three we used for the bulk of this analysis, were able to accurately align reads to the *Mup-ps22*. Yet, in the real Ion Torrent data, we saw a great deal of heterogeneity in each aligner’s ability to map reads to this gene. In addition to this gene/pseudogene pair, we also saw a similar finding with *Serpina3c* and *Serpina3i*, two members of the *Serpina3* gene family (Additional file 22 - panel A). Since these genes are induced by IL-1β treatment (Additional file 22 - panel B), we also saw the choice of aligner and platform affected the fold-change expression we calculated between the two experimental conditions (Additional file 22 - panel C). Taken together, these findings suggest that the differing coverage patterns of *Mup20* and *Mup-ps22* (as well as the *Serpina3* genes) between the Illumina and Ion Torrent data is not simply a function of the aligner choice, but rather an interaction between both the aligner and the sequencing platform.

## Discussion

Both Illumina and Ion Torrent provide alternative methods for researchers to study RNA at the sequence level. Here we compare the performance of these two technologies by investigating their ability to detect differentially expressed genes and pathways in a treatment/control experimental paradigm involving the effect of IL-1β treatment on the mouse liver. We found very high concordance between both of these technologies in terms of gene-level read counts, which is in agreement with the previous comparison studies [2, 8]. Additionally, we detected similar sets of differentially expressed genes in both sequencing platforms, and ultimately both Illumina and Ion Torrent data led to identical biological conclusions at the pathway level. In short, our results suggest a researcher would write the same paper, regardless of platform choice.

That being said, we did notice differences between the data from both platforms. Within the datasets generated by each aligner, we found 18–25% of the identified DEGs were platform-specific. These differences are comparable to those from previous studies using UHRR samples where biological variability was not even a factor [33]. It is likely the majority of these platform-specific DEGs are the results of the technical variability arising from differences in the library preparation and sequencing technologies of both platforms. This hypothesis is further supported by two observations: 1) most of the platform-specific DEGs are close to our threshold for statistical significance, and 2) 15–30% of the platform-specific DEGs identified using only one of the alignment algorithms were identified in both platforms when considering all three alignment algorithms together.

These observations led to the surprising finding that there appears to be an interaction between alignment algorithm and platform that affects the ability to detect absolute and differential gene expression. Others have noticed the impact of aligner choice on downstream analysis within a single sequencing platform [12, 34]. Here not only do we see these effects as well, we also observe that the impact of aligner choice is different depending upon whether we are using data derived from Illumina or Ion Torrent. Given that several of these aligners were developed prior to the introduction of the Ion Torrent platform, it is possible some of these interactions are due to the underlying assumptions of these algorithms, which are based largely on Illumina data. As a result, it may be possible to reduce the effects of this interaction through careful tuning of the alignment algorithm parameters to optimize for Ion Torrent. Alternatively, this platform/aligner interaction may prove to increase the utility of these RNA-Seq technologies. For both platforms, researchers already use different library preparation methods to study small RNAs and non-coding transcripts. Perhaps particular combinations of alignment software and sequencing platform may be better suited for interrogating specific genomic or transcriptional phenomena, like gene/pseudogene pairs or fusion transcripts.

## Conclusions

Taken together, our results suggest that while researchers may be able to modulate their ability to detect different transcripts through careful selection of sequencing platform and alignment algorithm, on the whole Illumina and Ion Torrent are equally suited to the task of expression analysis in treatment/control experiments.

## Methods

### Animal care, tissue collection, and RNA extraction

Wild-type, twelve-week old male C57/B6J mice were acquired from Jackson Labs (Bar Harbor, Maine, USA). Ten mice were treated with 20 μg/Kg of IL-1β (Sigma-Aldrich, catalog no. I9401; *n* = 5) or saline (*n* = 5; referred to as untreated in this manuscript), via intraperitoneal injection. Four hours after treatment, the mice were euthanized through carbon dioxide induced asphyxiation and liver samples were dissected and snap-frozen in liquid nitrogen. RNA was extracted from the liver tissue using TRIzol (ThermoFisher Scientific, catalog no. 15596018) and RNeasy Mini Kit (Qiagen, catalog no. 74104), according to manufacturers’ protocols. After extraction, total RNA was analyzed on a BioAnalyzer 2100 (Agilent) to check for integrity. All procedures were approved and carried out in accordance with the Institutional Animal Care and Use Committee of the University of Pennsylvania.

### Illumina library preparation and sequencing

200 ng of total RNA from each liver sample was prepared for Illumina sequencing according to the manufacturer’s protocol using the TruSeq stranded mRNA Sample Preparation Kit (Illumina, catalog no. RS-122-2103). Following preparation, library qualities were assessed using a Bioanalyzer 2100. Libraries from all samples were pooled together and sequenced using an Illumina HiSeq 2500 (125 bp paired-end reads).

### Ion torrent library preparation and sequencing

Total RNA was poly-A selected using the Dynabeads mRNA Direct Micro Purification kit (ThermoFisher, catalog no. 61021), according to manufacturer’s protocol. About 100 ng of poly-A RNA were used to prepare strand-specific barcoded RNA libraries with the Ion Total RNA-Seq kit v2.0 (ThermoFisher Scientific, catalog no. 4475936) following manufacturer’s protocol. The library qualities were checked by running on a BioAnalyzer 2100 and the concentrations were determined from the analysis profiles. Ten barcoded libraries were pooled together on an equimolar basis and run using three PIv3 chips on an Ion Torrent Proton using HiQ chemistry.

### RNA-Seq data alignment and normalization

We aligned fastq files from both platforms using STAR v2.5.1b [14], GSNAP release 2015–12-31 (v8) [13], or a combination of STAR and Bowtie2 v2.2.9 [16]. For the STAR + Bowtie2 combination, we first aligned reads to the genome using STAR and extracted all unmapped reads from the resulting BAM files. Next, we used Bowtie2 to align all of these unmapped reads to the reference genome. Lastly, we used custom perl scripts to merge the Bowtie2 alignments with the STAR alignment, replacing entries for the unaligned reads with the mapping information from Bowtie2. We mapped reads to the mm9 version of the reference genome (downloaded from the UCSC genome browser [35]) for all alignment algorithms. Also, we provided GSNAP and STAR with gene models from the Ensembl v67 genome annotation [36]. See the Additional file 1: Supplementary Methods for the full commands we used for each step in the alignment.

To normalize the data within a given platform/aligner combination, we used the **P**ipeline **O**f **R**NA-Seq **T**ransformations v0.8.1-beta (PORT) [19]. PORT is an implementation of the read re-sampling approach for normalization proposed by Li and Tibshirani [37]. Briefly, PORT filters out potential confounding factors like reads mapping to rRNA sequences and mitochondrial DNA. Next, PORT determines the input dataset with the fewest number of gene-mapping reads and re-samples all datasets to have the same number of reads, thus accounting for batch effects and differences in sequencing depth between samples. As a result, the normalized BAM and coverage files generated by PORT are directly comparable to each other. In addition to normalization, we also used PORT to quantify the normalized, gene-level read counts for each of our datasets. For quantification, we used the gene models from the Ensembl v67 annotation.

### RNA-Seq data analysis

We performed the majority of our quantification and differential expression analyses of the PORT quantification results in R. Before any other analyses, we filtered out all genes with low expression. Briefly, we only retained those genes with at least five mapped reads, in five of the ten total samples. This reduced the original 37,681 input genes to ~15,000 detected genes in each of the samples. We used this set of expression-filtered genes for the remainder of our analyses. To identify genes with differential expression between the untreated and IL-1β treatment conditions, we performed a two-sided Mann-Whitney *U* test [21], as implemented by the *wilcox.test* function in R. Lastly, we accounted for multiple testing using a Benjamini-Hochberg correction [22], as implemented by the *p.adjust* function in R. For the purposes of our analyses, we identified significantly differentially-expressed genes as those with Benjamini-Hochberg *q*-values <0.05. We also used the *limma* (v3.28.17) [23] software package to perform a parallel differential expression analysis. All further analyses and visualization of the data were performed using custom R scripts.

### qPCR

Real-time PCR for *Sperina3* genes was performed using ABI Taqman primers (ThermoFisher Scientific) and reagents on an ABI Prizm 7500 thermocycler according to manufacturer’s instructions: *Serpina3c* (ThermoFisher catalog no. 4331182; Mm00434669_m1) and *Serpina3i* (ThermoFisher catalog no. 4331182; Mm01612859_m1). Since *Mup-ps22* required custom primers, real-time PCR for mouse urinary protein genes was performed using SYBR reagents on the same instrument: *Mup20* (IDT PrimeTime qPCR Primer Assays in Tubes; Mm.PT.58.14054833), *Mup-ps22* (custom primers ordered from Sigma-Genosys; sequences in Additional file 1: Supplementary Methods). All mRNA measurements were normalized to *Gapdh* mRNA levels (Taqman assay - ThermoFisher catalog no. 4331182; Mm99999915_g1; SYBR assay - custom primers ordered from Sigma-Genosys; sequences in Additional file 1: Supplementary Methods).

### Ingenuity pathway analysis

To identify pathways enriched among our lists of DEGs, we used Ingenuity Pathway Analysis (IPA, Qiagen) [24]. We began by uploading the full tables of our Mann Whitney DEG results to the IPA server. These tables included the following information for each combination of platform and aligner: log_2_ fold-change differences between untreated and IL-1β treated samples, the *p*-values from the DEG test, and the multiple-testing corrected *q*-values. Next, we ran the IPA core analysis separately on the gene lists from each platform/aligner combination. For the core IPA analyses we identified DEGs with the following cutoffs: *q*-values <0.05 and absolute log2 fold-change values >1. For the purposes of enrichment tests, we used the list of all detected genes (i.e. our full IPA input tables) as the background set of genes. Aside from these changes, we used the default parameters for our IPA analyses.

### Down-sampling analysis and multiple aligner comparisons across both platforms

(See Additional file 1: Supplementary Methods for full details).

## Additional files

Additional file 1: Supplementary Methods. Supplementary information on the alignment command-line parameters, down-sampling analysis, RNA-Seq simulations, and custom qPCR primers for *Mup-ps22* a﻿nd *Gapdh*. (DOCX 21 kb)

Additional file 2: Figure S1. Alignment statistics. Bargraphs displaying the percentage of reads that either aligned uniquely (blue), aligned to multiple loci (green), or did not align (red) in each sample. These results are displayed for all combinations of platform and aligner. (PDF 137 kb)

Additional file 3: Table S1. Alignment metrics for all samples. For each sample, the following metrics are listed for each combination of platform and alignment algorithm: total number of reads (Ion Torrent) or read pairs (Illumina), percentage of uniquely-aligned reads, percentage of multimapped reads, percentage of unaligned reads, percentage of uniquely-aligned reads aligned to gene regions, percentage of multimapped reads aligned to gene regions, percentage of uniquely-aligned reads aligned to exonic regions, percentage of multimapped reads aligned to exonic regions, percentage of uniquely-aligned reads aligned to intronic regions, percentage of multimapped reads aligned to intronic regions, percentage of uniquely-aligned reads aligned to intergenic regions, and percentage of multimapped reads aligned to intergenic regions. (XLSX 17 kb)

Additional file 4: Figure S2. Read length distribution for Ion Torrent data. Read lengths were derived from the raw input files for each sample. (PDF 299 kb)

Additional file 5: Table S2. Ion Torrent read length statistics. The max, min, mean, and standard deviations of the read lengths in the Ion Torrent data, for each sample. (XLSX 9 kb)

Additional file 6: Figure S3. Read count comparison between platforms. Scatterplots comparing the gene-level read counts between Illumina (x-axis) and Ion Torrent (y-axis). Results are displayed for all samples, across all three alignment algorithms. Both axes are scaled to log10 of the PORT-normalized read counts. (PDF 344 kb)

Additional file 7: Table S3. Spearman correlation coefficients between Illumina and Ion Torrent read counts. Spearman correlation coefficients between Illumina and Ion Torrent read counts for each sample and aligner. (XLSX 11 kb)

Additional file 8: Figure S4. Mitochondrial and ribosomal content. Bargraphs displaying the percentage of reads that aligned to mitochondrial DNA (top; ChrM), or to ribosomal RNA sequences (bottom). Samples colored by treatment group (IL-1β = orange; untreated = blue). (PDF 86 kb)

Additional file 9: Table S4. Read count and differential expression results for all combinations of platform/aligner. Gene-level read counts from each sample, *p*-values from the Mann-Whitney *U* tests and *limma* for differential expression, Benjamini-Hochberg-corrected *q*-values. (XLSX 13131 kb)

Additional file 10: Figure S5. DEG concordance between platforms as a function of read depth. Line graph displaying the concordance (DEGS identified by both platforms/total number of DEGs) at varying levels of read depth. The regression line generated by the *glm* function in R is displayed in blue. (PDF 81 kb)

Additional file 11: Figure S6. Differential expression analysis with *limma.* Within each combination of platform and aligner, differentially-expressed genes (DEGs) were identified using the *limma* software package. Genes with BH *q*-values <0.05 were identified as differentially expressed. A) The overlap in DEGs between Illumina and Ion Torrent for each aligner. B) The overlaps in DEGs identified in each platform by *limma* or Mann-Whitney, for each aligner. C) MA plots for every combination of platform and aligner. Within each aligner, genes are colored according to the platform in which they were identified by *limma* as DEGs. (PDF 573 kb)

Additional file 12: Figure S7. Fold-change comparison between platforms. Scatterplots comparing the log2 fold-change values of differentially expression genes in the Illumina (x-axis) and Ion Torrent (y-axis) datasets, for each alignment algorithm. Within each aligner, genes are colored according to the platform in which they were identified as DEGs. For those DEGs with zero expression in the IL-1β or untreated condition, a pseudo-count of 1 was added to both the numerator and denominator for the fold-change calculation. (PDF 336 kb)

Additional file 13: Table S5. Spearman and Pearson correlation coefficients for Illumina vs Ion Torrent fold-change comparison. Spearman and Pearson correlation coefficients between Illumina and Ion Torrent log2 fold-change values, within each alignment algorithm. Separate correlation coefficients were calculated for DEGs identified by both platforms, by Illumina only, and by Ion Torrent only. (XLSX 9 kb)

Additional file 14: Table S6. Significant results from Ingenuity Pathway Analysis -- Canonical Pathways. IPA results from the canonical pathways analysis for each combination of platform and aligner. Table lists pathway names, enrichment *p*-values, z-scores, and the list of DEGs for each pathway. (XLSX 35 kb)

Additional file 15: Table S7. Top 10 results from Ingenuity Pathway Analysis -- Upstream Regulators. IPA results from the upstream regulators pathway analysis for each combination of platform and aligner. Table lists the identities of the upstream regulators, predicted activation state, *p*-value for DEG overlap with regulator targets, list of DEGs for each upstream regulator. (XLSX 19 kb)

Additional file 16:Table S8. The number of platform-specific genes detected by aligner. Numbers of platform-specific genes detected in each aligner as a function of the mean gene-level read counts across all samples. Also lists the number of DEGs among the platform-specific genes. (XLSX 11 kb)

Additional file 17: Figure S8. Read depth for platform-specific genes. Distributions of read counts for platform-specific genes are displayed for all samples, across all three alignment algorithms. The majority of platform-specific genes have less than 50 reads, so the graphs’ x-axes are limited to the [0, 50] range for display purposes. (PDF 1099 kb)

Additional file 18: Figure S9. GC-content of DEGs. Density plots, for each aligner, of the % GC content for DEGs identified by both platforms (red), Illumina only (blue), Ion Torrent only (green), and non-DEGs (black). (PDF 99 kb)

Additional file 19: Table S9. Ensembl biotypes of genes detected by both platforms, or exclusively by one platform. For each aligner, lists the number of and percent of detected genes for each ensembl biotype. These numbers are broken down by those genes detected in both platform, only in Illumina data, and only in Ion Torrent. (XLSX 12 kb)

Additional file 20: Figure S10. qPCR results for *Mup20* and *Mup-ps22.* Bargraphs display average expression across samples in each treatment group. *Gapdh* expression is used as the endogenous control. Error bars display the squared-error of the mean (SEM). (PDF 16 kb)

Additional file 21: Figure S11. Using a full dataset to examine platform/aligner interaction. For each sequencing platform, full fastq files from sample 9574 (untreated) were re-aligned using STAR + Bowtie2 and the twelve most popular aligners, according to a survey of the literature. This figure displays the number of uniquely-mapped (top) and multimapped (bottom) reads aligned to *Mup20* or *Mup-ps22*. (PDF 110 kb)

Additional file 22: Figure S12. Using simulated data to examine platform/aligner interaction. For each sequencing platform, all reads aligned by GSNAP, or STAR + Bowtie2 to *Serpina3c* or *Serpina3i* across all samples were extracted. These data were re-aligned using STAR + Bowtie2 and the twelve most popular aligners, according to a survey of the literature. Additionally, simulated RNA-Seq reads were generated from both of these genes. A) The average number of uniquely-mapped (top) and multimapped (bottom) reads aligned to each gene across the untreated and IL-1β-treated samples. B) qPCR results for *Serpina3c* and *Sperina3i*. C) Log_2_ fold-change differences between the average expression in the IL-1β-treated and untreated samples. (PDF 269 kb)

## Abbreviations

chrM: Mitochondrial Chromosome
DEG: Differentially Expressed Gene
DGE: Differential Gene Expression
IPA: Ingenuity Pathway Analysis
PORT: Pipeline Of RNA-Seq Transformations
RNA-Seq: RNA-Sequencing
rRNA: Ribosomal RNA
UHRR: Universal Human Reference RNA
