# Supplementary material for: A comparison of Illumina and Ion Torrent sequencing platforms in the context of differential gene expression
Source: BMC Genomics. 2017 Aug 10;18:602. doi: 10.1186/s12864-017-4011-0 (PMC5553782; doi:10.1186/s12864-017-4011-0)
Supplement: Supplementary file 1 — Supplementary Methods. Supplementary information on the alignment command-line parameters, down-sampling analysis, RNA-Seq simulations, and custom qPCR primers for Mup-ps22 a﻿nd Gapdh. (DOCX 21 kb) [file 12864_2017_4011_MOESM1_ESM.docx]

**Supplementary Methods**

**GSNAP alignment parameters**

For both Illumina (paired-end) and Ion Torrent data (single-end):

gsnap

-A sam

-K 50

--distant-splice-identity=1.0

-E 100

--max-mismatches 15

--indel-penalty 1

--gmap-min-match-length 10

--pairexpect 285 #This parameter is only used for the Illumina data

--merge-distant-samechr

--ordered

--novelsplicing 1

--use-splicing mm9_ens_v67.splicesites

--add-paired-nomappers

--gunzip

--nthreads 15

--batch 5

--expand-offsets 1

**STAR alignment parameters**

For both Illumina (paired-end) and Ion Torrent data (single-end):

STAR

--runMode alignReads

--runThreadN 4

--outSAMunmapped Within KeepPairs

--outFilterMismatchNmax 33

--seedSearchStartLmax 33

--alignSJoverhangMin 8

**Bowtie2 alignment parameters**

For both Illumina (paired-end) and Ion Torrent data (single-end):

bowtie2

--local

--very-sensitive-local

-q

--reorder

**Down-sample reads and perform differential expression analysis**

To examine the effect of coverage depth on the overlap between the two platforms, we began with the normalized SAM files generated by PORT. We extracted a list of unique read IDs (first column) from each SAM file. Next, we generated down-sampled read lists by randomly sampling 90%, 80%, 70%, 60%, and 50% of the read IDs from these files. To prepare down-sampled SAM files, we filtered the normalized SAM files using the lists of down-sampled read IDs prepared above. The end result was five additional, normalized SAM files for each sample, which contained decreased read depths relative to the original samples. We then quantified gene-level expression from each of these down-sampled files using the quantification scripts included with PORT. We already know that increasing coverage depth increases the total number of reads detected. To focus specifically on the impact of coverage depth on the ability to detect DEGs, we limited our analyses just to those genes that were detected across all down-sampled data. Lastly, we repeated our differential expression analysis using the Mann-Whitney *U* test (as described in the main *Methods* section) at each level of coverage. To assess the overlap between the two platforms, we calculated the concordance at each coverage level. Here we define concordance as the number of DEGs identified by both platforms divided by the total number of DEGs.

**Extract all reads aligning to *Mup20*, *Mup-ps22*, *Serpina3c*, and *Serpina3i***

In order to compare the effect of platform and aligner on the measured read depths from *Mup20* (ENSMUST00000074018), *Mup-ps22* (ENSMUST00000121611), *Serpina3c* (ENSMUST00000085050), and *Serpina3i* (ENSMUST00000109958), we first need to extract all reads that align to these loci. Referring to the Ensembl gene models, we determined the following spans for each of the four genes: chr4:61711267-61715192 (*Mup20*), chr11:54937197-54937590 (*Mup-ps22*), chr12:105384046-105392673 (*Serpina3c*), and chr12:105501202-105507821 (*Serpina3i*). We performed the following steps separately for the data from each sample and platform. First, we used samtools (v1.3.1) [1] to extract all read alignments overlapping these regions from the PORT-normalized SAM files produced from STAR, GSNAP, and STAR+Bowtie2 output. This includes both uniquely-aligned reads and multimappers that had at least one alignment to these loci. Next, we pooled the alignments generated by each aligner, and extracted a unique list of read IDs (column 1 in a SAM alignment) for all reads aligned to our regions of interest. Lastly, we used this list of read IDs to extract the corresponding entries from the raw fastq files. So following these steps, for each combination of sample and aligner, we have a fastq file containing those reads/read pairs that were aligned to one of our four genes of interest by STAR, GSNAP, or STAR+Bowtie2. We used these fastq files as input for the thirteen aligner comparison described below.

**Thirteen aligner comparison**

We used twelve of the aligners benchmarked by a previous study from our lab [2], plus the STAR+Bowtie2 alignment strategy, to re-align those reads mapped by STAR, GSNAP, or STAR+Bowtie2 to *Mup20*, *Mup-ps22*, *Serpina3c*, and *Serpina3i* (described above). We also re-aligned the full fastq files for sample 9574 (untreated) using all thirteen aligners. The name, reference, version, and extra command line parameters used for each of these tools are listed in the following table:

| **Aligner** | **Reference** | **Version** | **Extra command line parameters** |
| --- | --- | --- | --- |
| ContextMap2 | Bonfert *et al*., 2015 [3]. | 2.6.0 | --noncanonicaljunctions –aligner_name bwa --verbose |
| CRAC | Philippe *et al*., 2013 [4]. | 2.4.0 | -k 22 -no-ambiguity  Illumina & Simulated: --reads-length 125 |
| GSNAP | Wu and Nacu, 2010 [5]. | 2015-9-29 | -A sam --merge-distant-samechr --ordered  --novelsplicing 1 --expand-offsets 1 |
| HISAT | Kim *et al*., 2015 [6]. | 0.1.6beta | --time --reorder -q |
| HISAT2 | Kim *et al*., 2015 [6]. | 2.0.0beta | --time --reorder -q |
| MapSplice2 | Wang *et al*., 2010 [7]. | 2.2.0 | --non-canonical-double-anchor |
| OLego | Wu *et al*., 2013 [8]. | 1.1.6 | --verbose |
| RUM | Grant *et al*., 2011 [9]. | 2.0.5_06 | --verbose  Ion Torrent: --min-length 25  --variable-length-reads  Illumina & Simulated: --preserve-names |
| SOAPSplice | Huang *et al*., 2011 [10]. | 1.10 | -f 2 -q 1  Illumina: -l 0 -I 182  Simulated: -l 0 -I 260 |
| STAR | Dobin *et al*., 2013 [11]. | 2.5.0a | --outSAMunmapped Within |
| STAR+Bwt2 | Blair, 2014 [12]. | 2.5.0a/2.2.9 | STAR: --outSAMunmapped Within  Bowtie2: --local --very-sensitive-local -q  --reorder |
| Subread | Liao *et al*., 2013 [13]. | 1.5.0 | --allJunctions --SAMoutput |
| TopHat2 | Kim *et al*., 2013 [14]. | 2.1.0 | Illumina: --mate-inner-dist 0  --mate-std-dev 52  Simulated: --mate-inner-dist 56  --mate-std-dev 50 |

Following alignment, we quantified gene-level expression using the featureCounts tool from the Subread package. We found similar quantification results using HTSeq and in-house quantification scripts, and elected to use featureCounts due to its speed and ease of use for aligning large batches of data. For each SAM file, we quantified gene counts using just the uniquely-aligned reads, and then repeated the quantification using all reads (+multimappers). Here are the commands we used for quantification: “featureCounts –C –R –p –s 2” (Illumina data), “featureCounts –C –R –s 1” (Ion Torrent data). For quantification including multimappers, add the “-M” argument to both commands.

**Simulated RNA-Seq reads**

We simulated RNA-Seq data using the BEERS software package [9], with Ensembl gene models for *Mup20* (ENSMUST00000074018), *Mup-ps22* (ENSMUST00000121611), *Serpina3c* (ENSMUST00000085050), and *Serpina3i* (ENSMUST00000109958). For the MUP genes we simulated *Mup20* expression at twice the level of *Mup-ps22*. For the SERPINA3 genes, we simulated *Serpina3c* expression at five times the level of *Serpina3i*. Both of these ranges were selected to roughly match the relative expression between these genes in the GSNAP-aligned Ion Torrent data, as these alignment results showed a middle-ground among the results of all thirteen aligners. When running BEERS, all error, intronic read, and polymorphism parameters were set to zero and the remaining parameters used default values. We aligned these simulated reads using each of the thirteen aligners as described above. For the simulated data, we used the following command to quantify the alignment results (this is due to the way the simulator generates strand-specific data): “featureCounts –C –R –p –s 1”. Again, to quantify the simulated data including multimappers, add the “-M” argument to the featureCounts command.

**Sequences for custom SYBR primers:**

*Mup-ps22*

Forward primer: CATCTGACAGAATTCTTTGGCCG

Reverse primer: TCGAGGCAGCAATTGGCATT

*Gapdh*

Forward primer: CATGGCCTTCCGTGTTCCT

Reverse primer: GCGGCACGTCAGATCCA

**References**

1. Li H, Handsaker B, Wysoker A, Fennell T, Ruan J, Homer N, et al. The Sequence Alignment/Map format and SAMtools. Bioinforma. Oxf. Engl. 2009;25:2078–9.

2. Baruzzo G, Hayer KE, Kim EJ, Di Camillo B, FitzGerald GA, Grant GR. Simulation-based comprehensive benchmarking of RNA-seq aligners. Nat. Methods. 2016;

3. Bonfert T, Kirner E, Csaba G, Zimmer R, Friedel CC. ContextMap 2: fast and accurate context-based RNA-seq mapping. BMC Bioinformatics. 2015;16:122.

4. Philippe N, Salson M, Commes T, Rivals E. CRAC: an integrated approach to the analysis of RNA-seq reads. Genome Biol. 2013;14:R30.

5. Wu TD, Nacu S. Fast and SNP-tolerant detection of complex variants and splicing in short reads. Bioinformatics. 2010;26:873–81.

6. Kim D, Langmead B, Salzberg SL. HISAT: a fast spliced aligner with low memory requirements. Nat. Methods. 2015;12:357–60.

7. Wang K, Singh D, Zeng Z, Coleman SJ, Huang Y, Savich GL, et al. MapSplice: accurate mapping of RNA-seq reads for splice junction discovery. Nucleic Acids Res. 2010;38:e178.

8. Wu J, Anczuków O, Krainer AR, Zhang MQ, Zhang C. OLego: fast and sensitive mapping of spliced mRNA-Seq reads using small seeds. Nucleic Acids Res. 2013;41:5149–63.

9. Grant GR, Farkas MH, Pizarro AD, Lahens NF, Schug J, Brunk BP, et al. Comparative analysis of RNA-Seq alignment algorithms and the RNA-Seq unified mapper (RUM). Bioinforma. Oxf. Engl. 2011;27:2518–28.

10. Huang S, Zhang J, Li R, Zhang W, He Z, Lam T-W, et al. SOAPsplice: Genome-Wide ab initio Detection of Splice Junctions from RNA-Seq Data. Front. Genet. 2011;2:46.

11. Dobin A, Davis CA, Schlesinger F, Drenkow J, Zaleski C, Jha S, et al. STAR: ultrafast universal RNA-seq aligner. Bioinforma. Oxf. Engl. 2013;29:15–21.

12. Blair K. Ion Proton RNA-Seq: in search of the best alignment method - Seven Bridges [Internet]. Seven Bridg. 2014 [cited 2016 Sep 4]. Available from: https://blog.sbgenomics.com/ion-proton-rna-seq-alignment/

13. Liao Y, Smyth GK, Shi W. The Subread aligner: fast, accurate and scalable read mapping by seed-and-vote. Nucleic Acids Res. 2013;41:e108.

14. Kim D, Pertea G, Trapnell C, Pimentel H, Kelley R, Salzberg SL. TopHat2: accurate alignment of transcriptomes in the presence of insertions, deletions and gene fusions. Genome Biol. 2013;14:R36.
