# Supplementary figures and images for: A comparison of Illumina and Ion Torrent sequencing platforms in the context of differential gene expression
Source: BMC Genomics. 2017 Aug 10;18:602. doi: 10.1186/s12864-017-4011-0 (PMC5553782; doi:10.1186/s12864-017-4011-0)

Percent of total reads

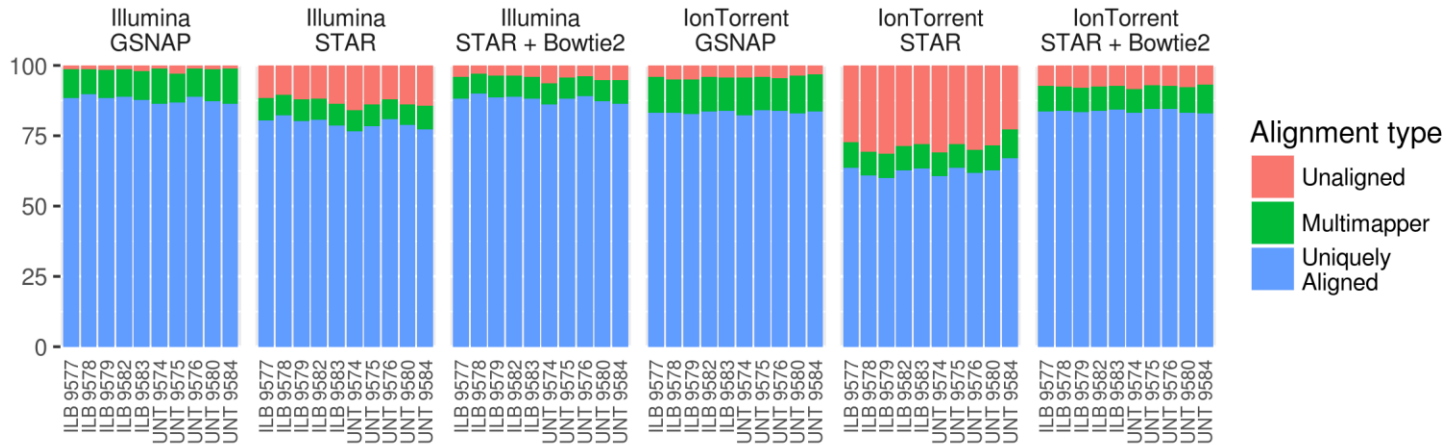

Supplement: Supplementary file 2 — Alignment statistics. Bargraphs displaying the percentage of reads that either aligned uniquely (blue), aligned to multiple loci (green), or did not align (red) in each sample. These results are displayed for all combinations of platform and aligner. (PDF 137 kb) [file 12864_2017_4011_MOESM2_ESM.pdf]

# Ion Torrent read length distribution

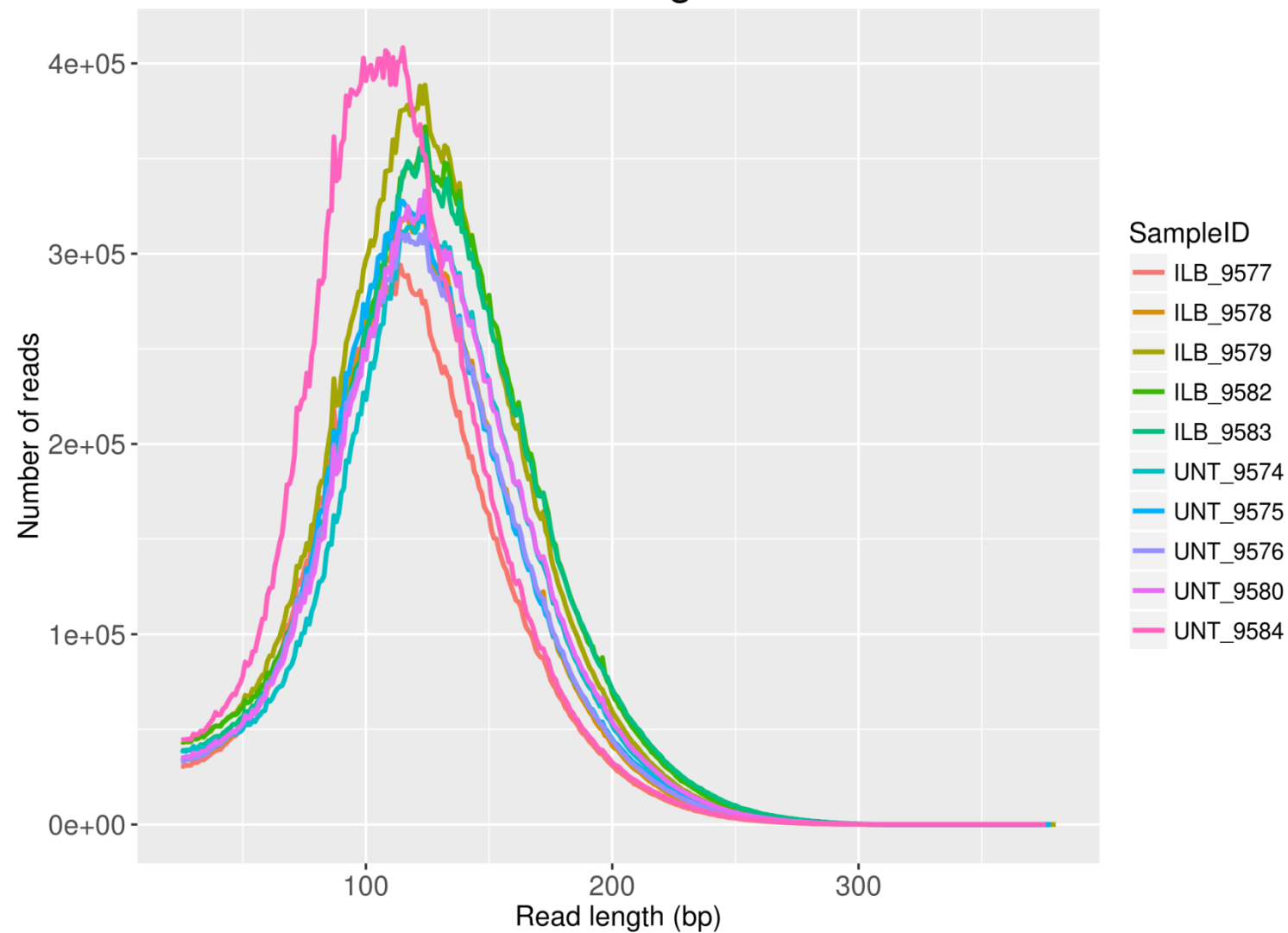

Supplement: Supplementary file 4 — Read length distribution for Ion Torrent data. Read lengths were derived from the raw input files for each sample. (PDF 299 kb) [file 12864_2017_4011_MOESM4_ESM.pdf]

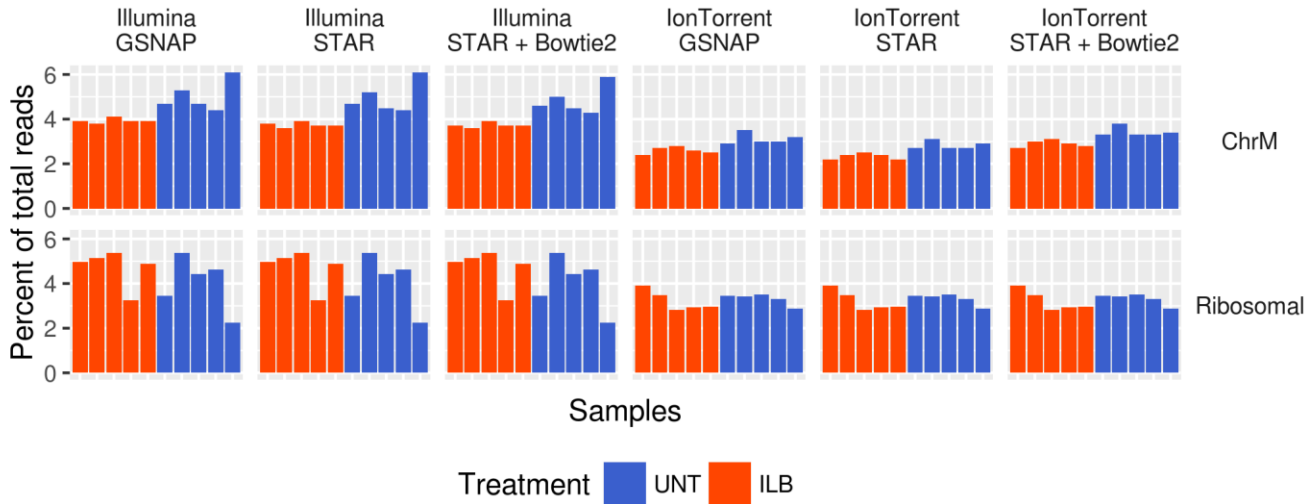

Supplement: Supplementary file 8 — Mitochondrial and ribosomal content. Bargraphs displaying the percentage of reads that aligned to mitochondrial DNA (top; ChrM), or to ribosomal RNA sequences (bottom). Samples colored by treatment group (IL-1β = orange; untreated = blue). (PDF 86 kb) [file 12864_2017_4011_MOESM8_ESM.pdf]

# DEG concordance between platforms as a function of read depth

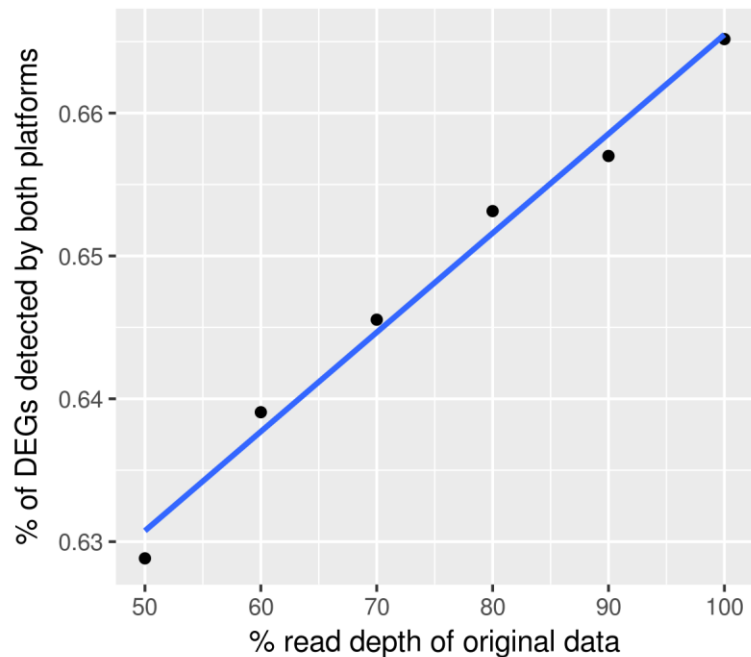

Supplement: Supplementary file 10 — DEG concordance between platforms as a function of read depth. Line graph displaying the concordance (DEGS identified by both platforms/total number of DEGs) at varying levels of read depth. The regression line generated by the glm function in R is displayed in blue. (PDF 81 kb) [file 12864_2017_4011_MOESM10_ESM.pdf]

**A**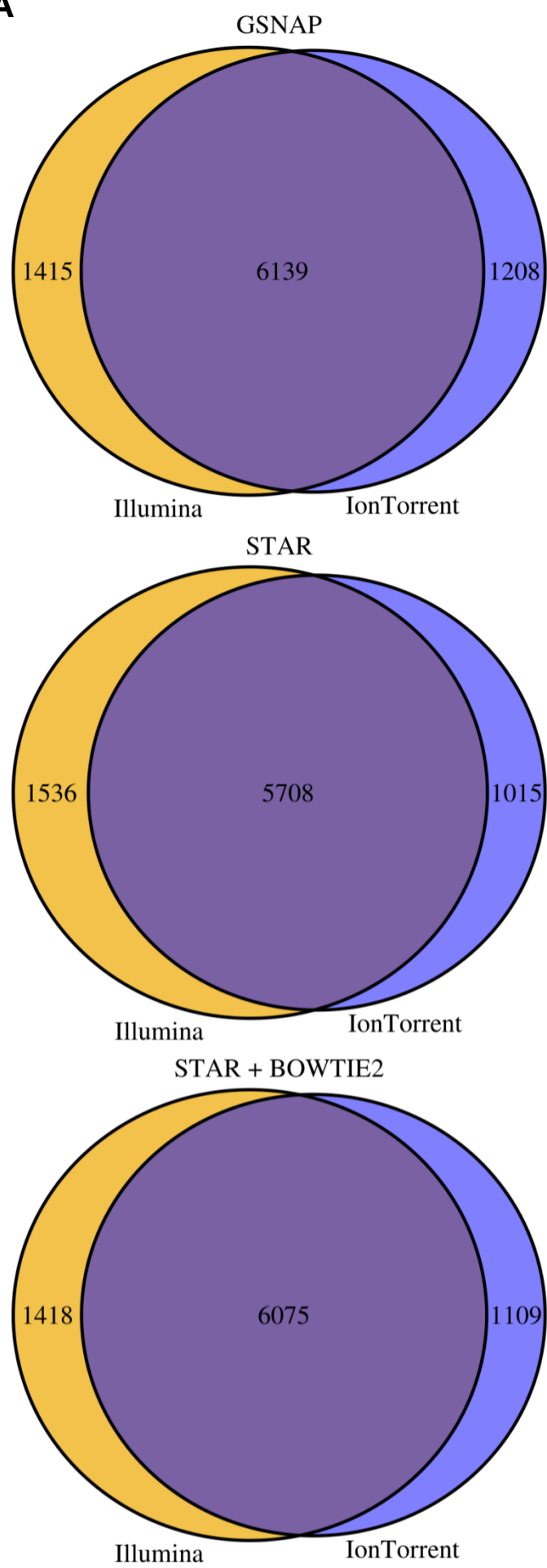**B**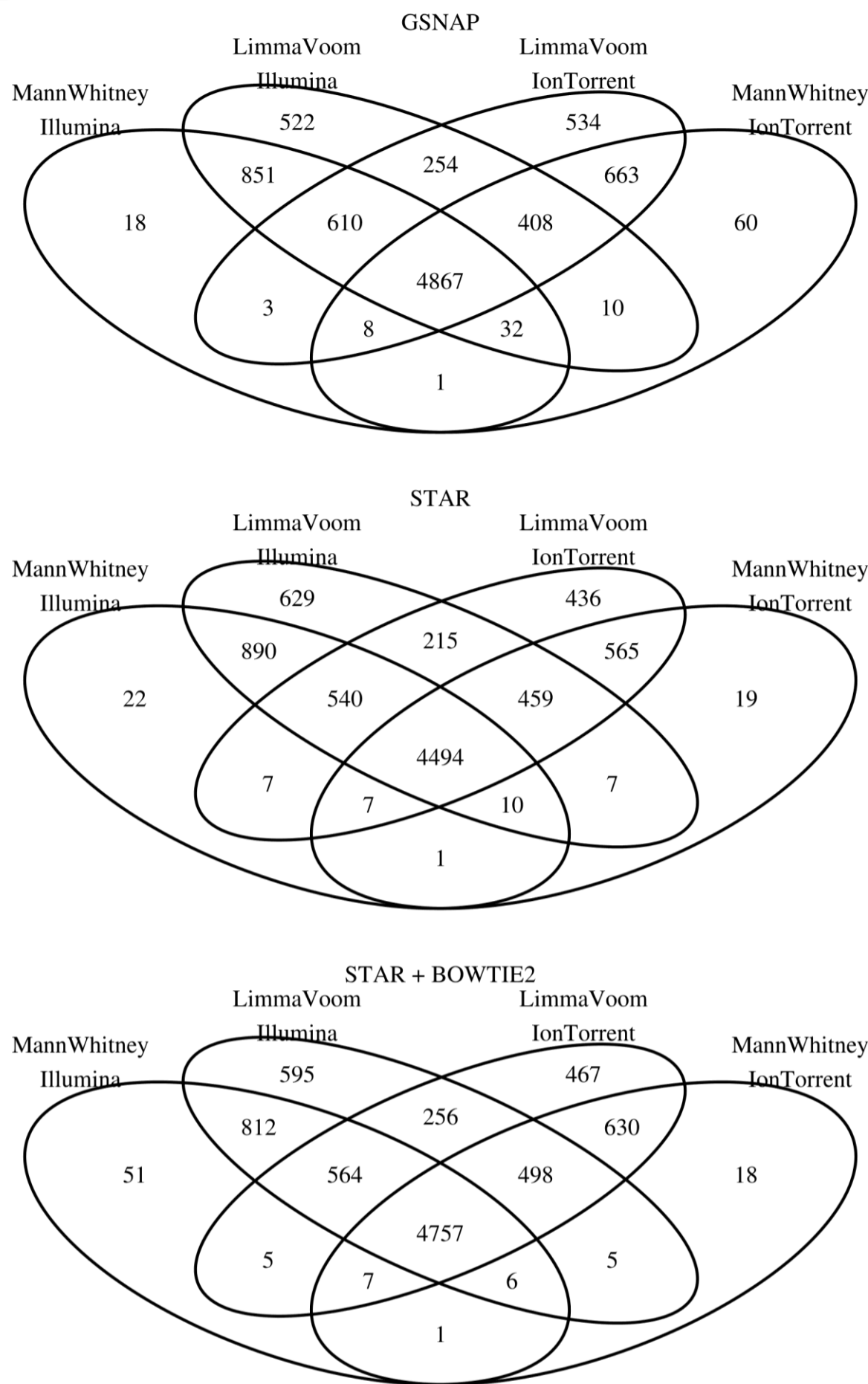**C**

MA plots (IL-1B / Untreated)

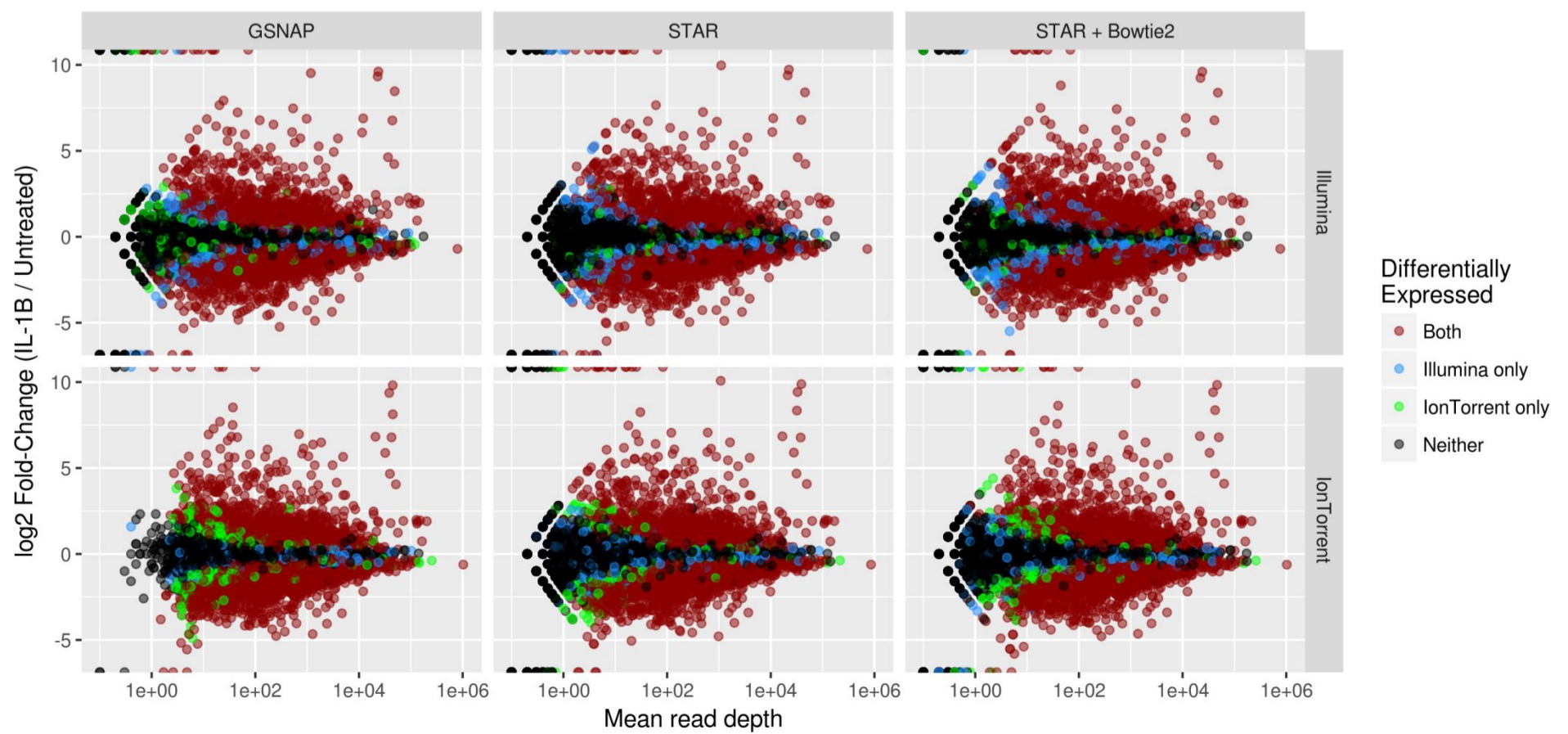

Supplement: Supplementary file 11 — Differential expression analysis with limma. Within each combination of platform and aligner, differentially-expressed genes (DEGs) were identified using the limma software package. Genes with BH q-values <0.05 were identified as differentially expressed. A) The overlap in DEGs between Illumina and Ion Torrent for each aligner. B) The overlaps in DEGs identified in each platform by limma or Mann-Whitney, for each aligner. C) MA plots for every combination of platform and aligner. Within each aligner, genes are colored according to the platform in which they were identified by limma as DEGs. (PDF 573 kb) [file 12864_2017_4011_MOESM11_ESM.pdf]

# Fold-change (ILB / UNT) comparison

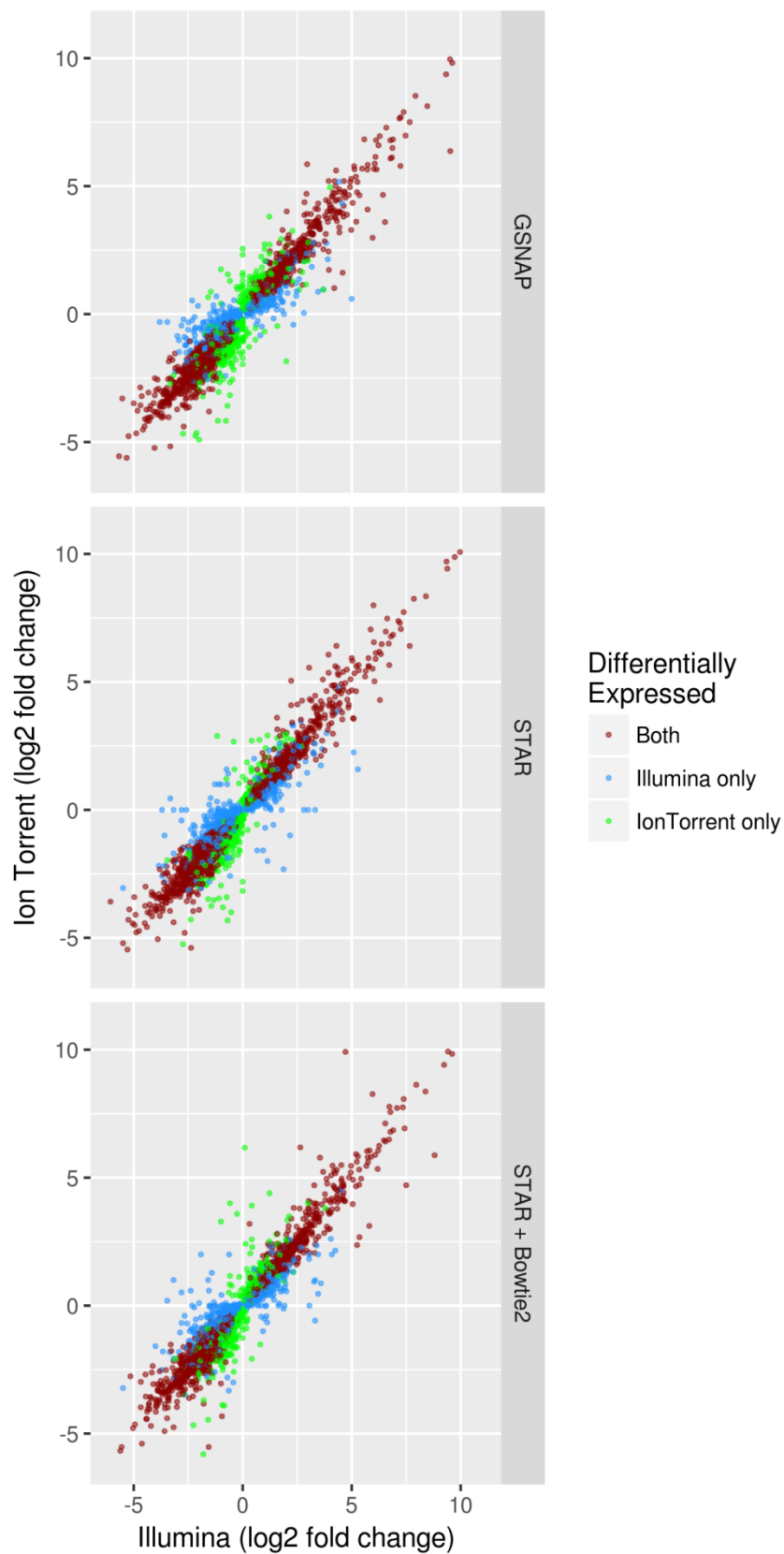

Supplement: Supplementary file 12 — Fold-change comparison between platforms. Scatterplots comparing the log2 fold-change values of differentially expression genes in the Illumina (x-axis) and Ion Torrent (y-axis) datasets, for each alignment algorithm. Within each aligner, genes are colored according to the platform in which they were identified as DEGs. For those DEGs with zero expression in the IL-1β or untreated condition, a pseudo-count of 1 was added to both the numerator and denominator for the fold-change calculation. (PDF 336 kb) [file 12864_2017_4011_MOESM12_ESM.pdf]

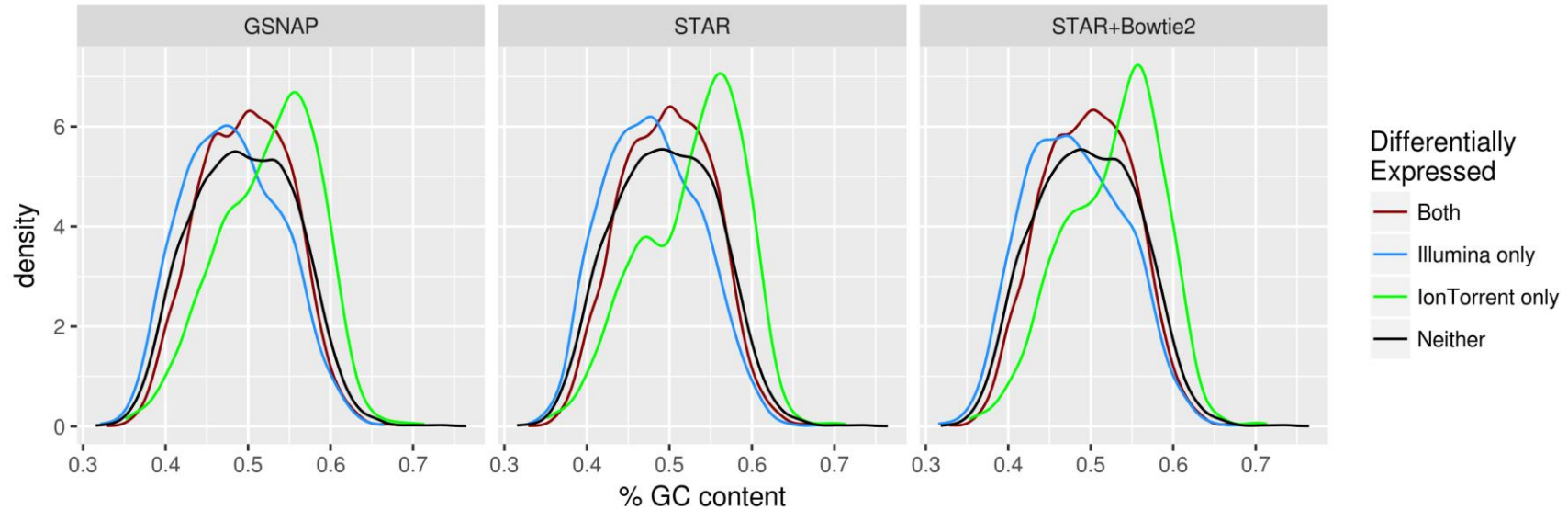

Supplement: Supplementary file 18 — GC-content of DEGs. Density plots, for each aligner, of the % GC content for DEGs identified by both platforms (red), Illumina only (blue), Ion Torrent only (green), and non-DEGs (black). (PDF 99 kb) [file 12864_2017_4011_MOESM18_ESM.pdf]

# qPCR

Mean expression (relative to *Gapdh*)

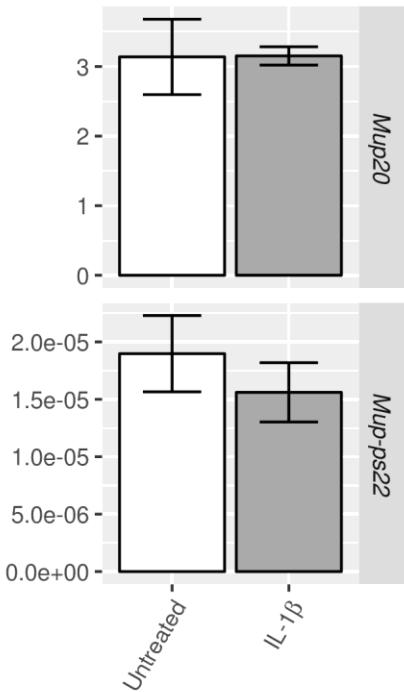

Supplement: Supplementary file 20 — qPCR results for Mup20 and Mup-ps22. Bargraphs display average expression across samples in each treatment group. Gapdh expression is used as the endogenous control. Error bars display the squared-error of the mean (SEM). (PDF 16 kb) [file 12864_2017_4011_MOESM20_ESM.pdf]

## Uniquely-mapped reads

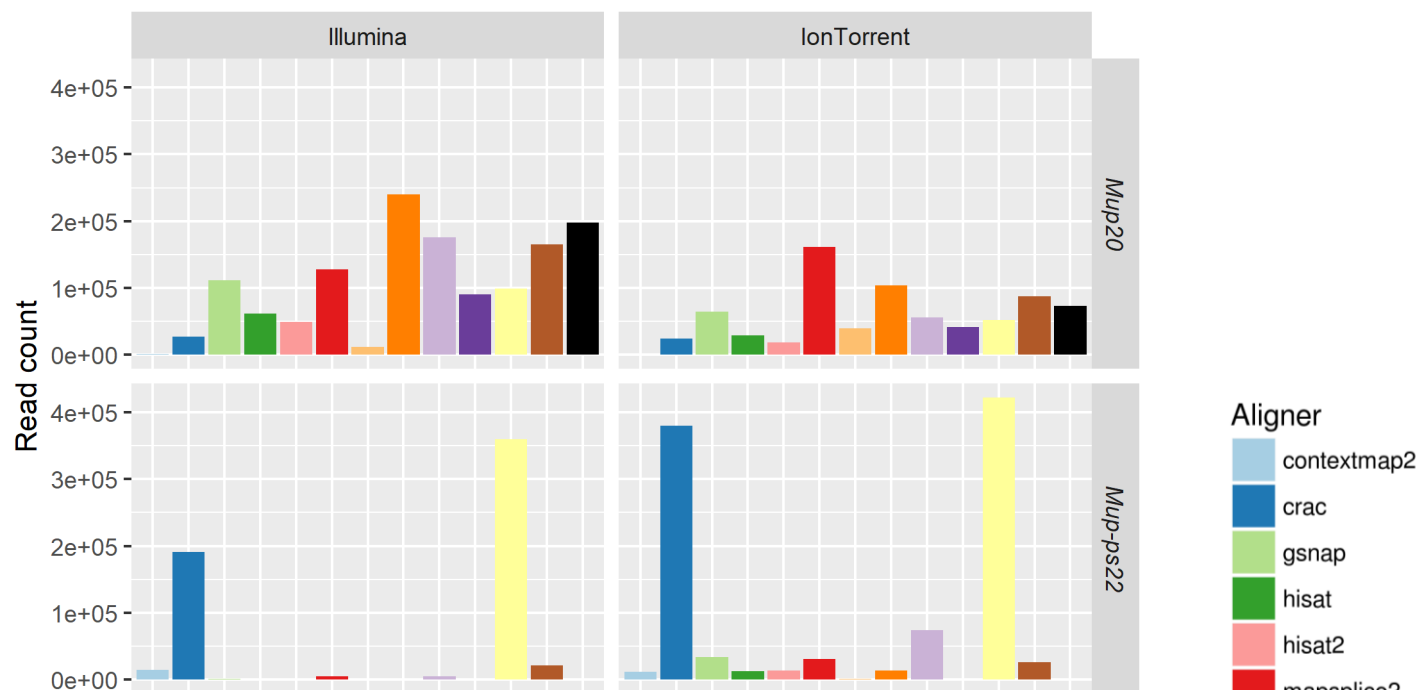

## Multi-mapped reads

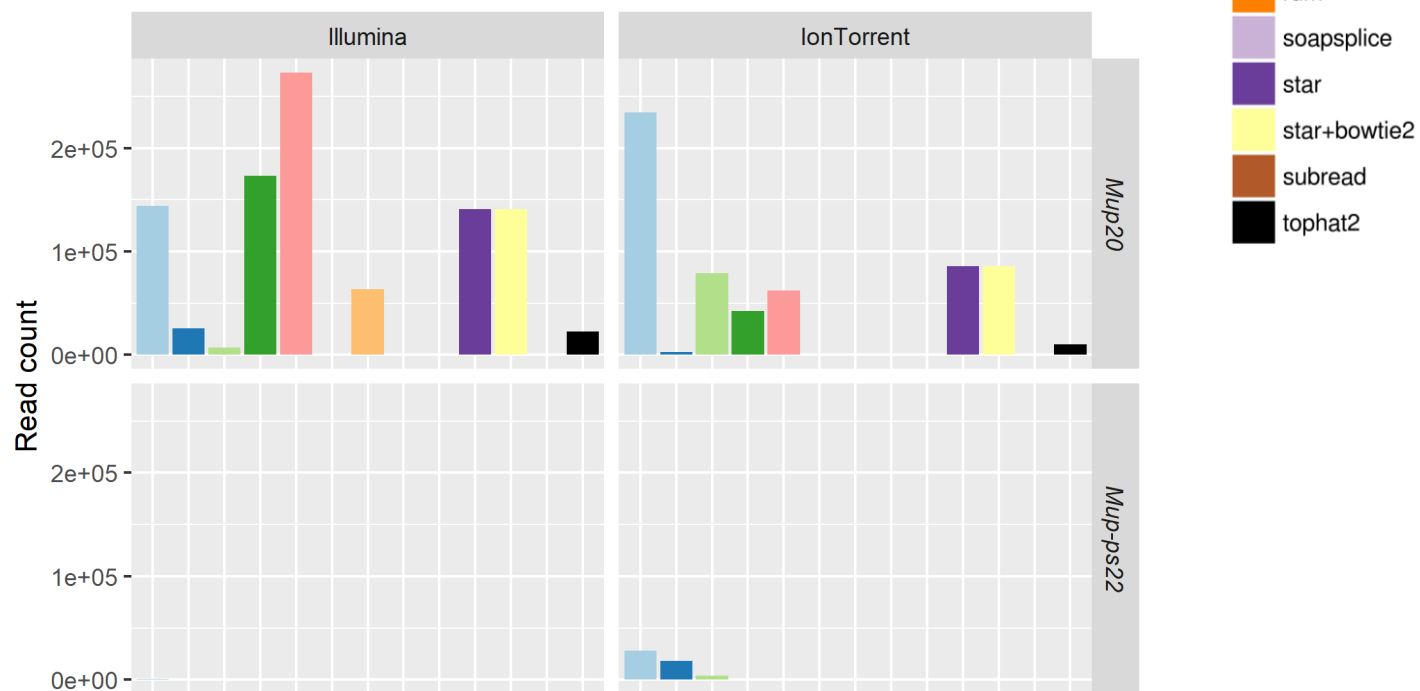

Supplement: Supplementary file 21 — Using a full dataset to examine platform/aligner interaction. For each sequencing platform, full fastq files from sample 9574 (untreated) were re-aligned using STAR + Bowtie2 and the twelve most popular aligners, according to a survey of the literature. This figure displays the number of uniquely-mapped (top) and multimapped (bottom) reads aligned to Mup20 or Mup-ps22. (PDF 110 kb) [file 12864_2017_4011_MOESM21_ESM.pdf]

**A**

### Uniquely-mapped reads

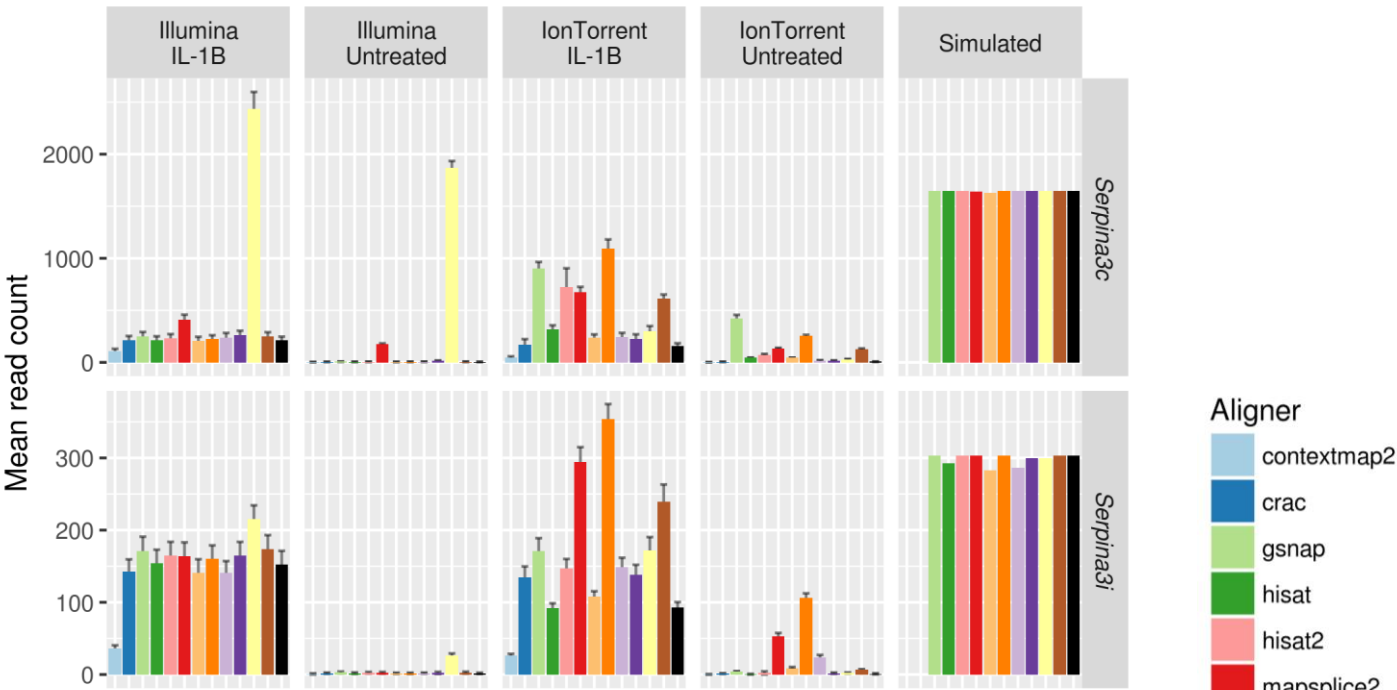

### Multi-mapped reads

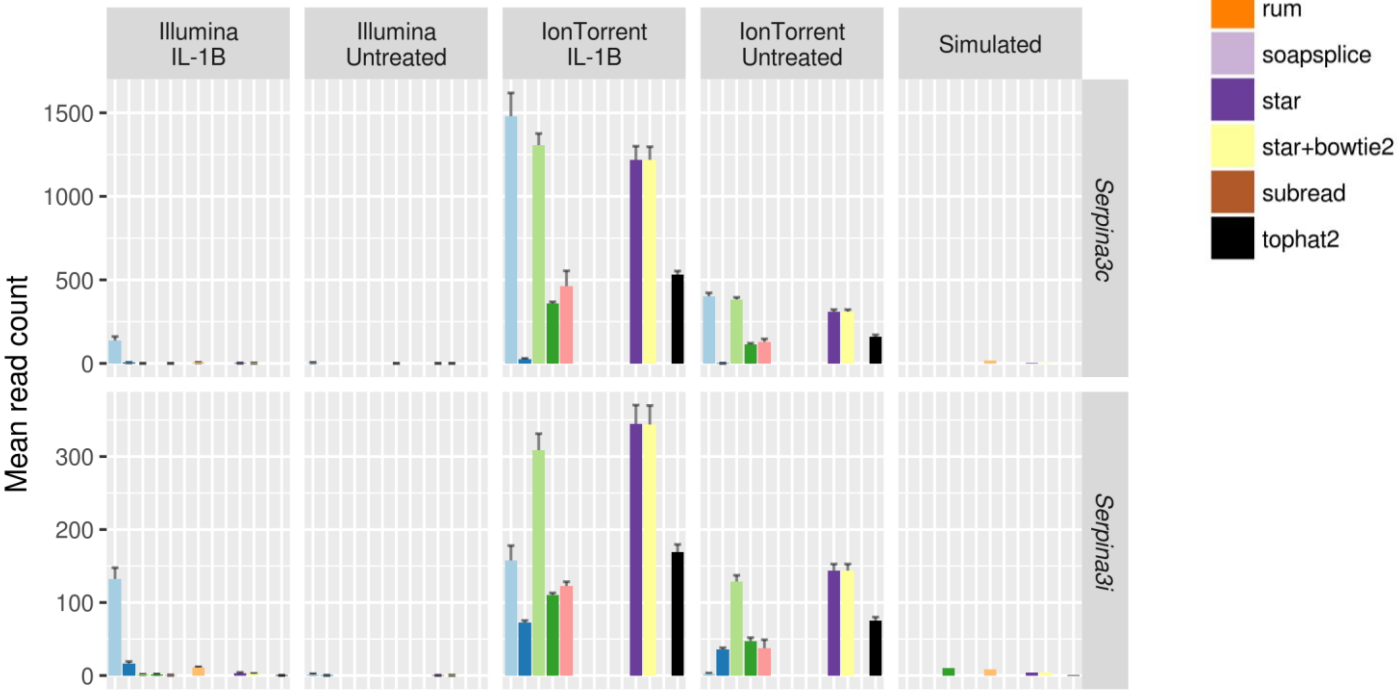

**B**

### qPCR

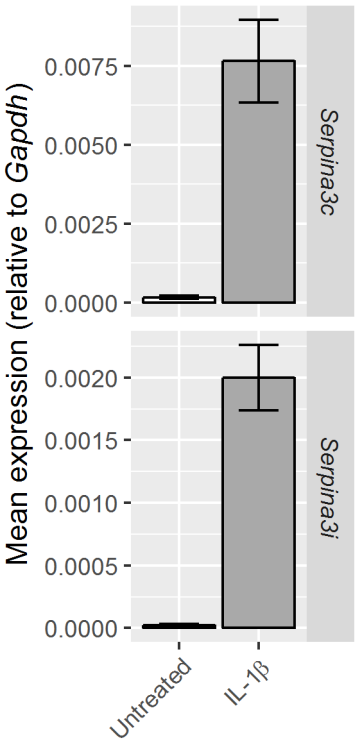

**C**

### Fold-change

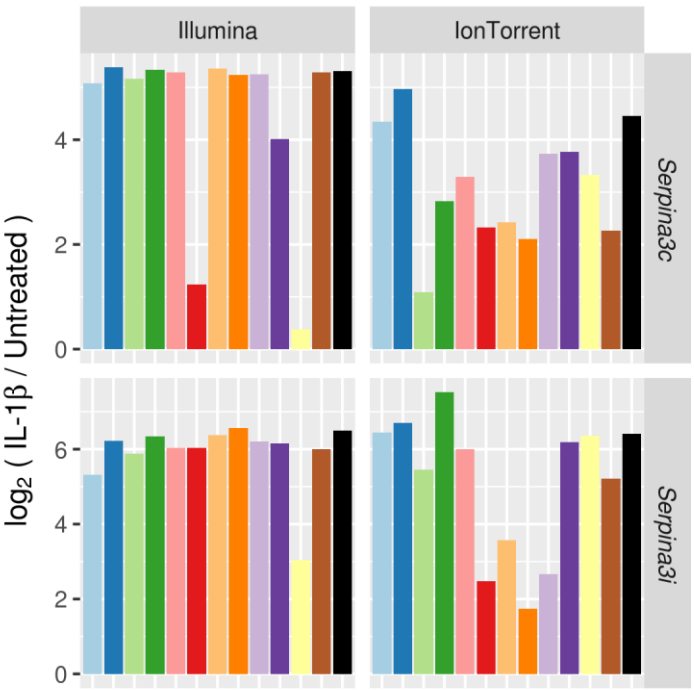

Supplement: Supplementary file 22 — Using simulated data to examine platform/aligner interaction. For each sequencing platform, all reads aligned by GSNAP, or STAR + Bowtie2 to Serpina3c or Serpina3i across all samples were extracted. These data were re-aligned using STAR + Bowtie2 and the twelve most popular aligners, according to a survey of the literature. Additionally, simulated RNA-Seq reads were generated from both of these genes. A) The average number of uniquely-mapped (top) and multimapped (bottom) reads aligned to each gene across the untreated and IL-1β-treated samples. B) qPCR results for Serpina3c and Sperina3i. C) Log2 fold-change differences between the average expression in the IL-1β-treated and untreated samples. (PDF 269 kb) [file 12864_2017_4011_MOESM22_ESM.pdf]
