# Supplementary material for: A comparison of Illumina and Ion Torrent sequencing platforms in the context of differential gene expression
Source: BMC Genomics. 2017 Aug 10;18:602. doi: 10.1186/s12864-017-4011-0 (PMC5553782; doi:10.1186/s12864-017-4011-0)
Supplement: Supplementary file 6 — Read count comparison between platforms. Scatterplots comparing the gene-level read counts between Illumina (x-axis) and Ion Torrent (y-axis). Results are displayed for all samples, across all three alignment algorithms. Both axes are scaled to log10 of the PORT-normalized read counts. (PDF 344 kb) [file 12864_2017_4011_MOESM6_ESM.pdf]

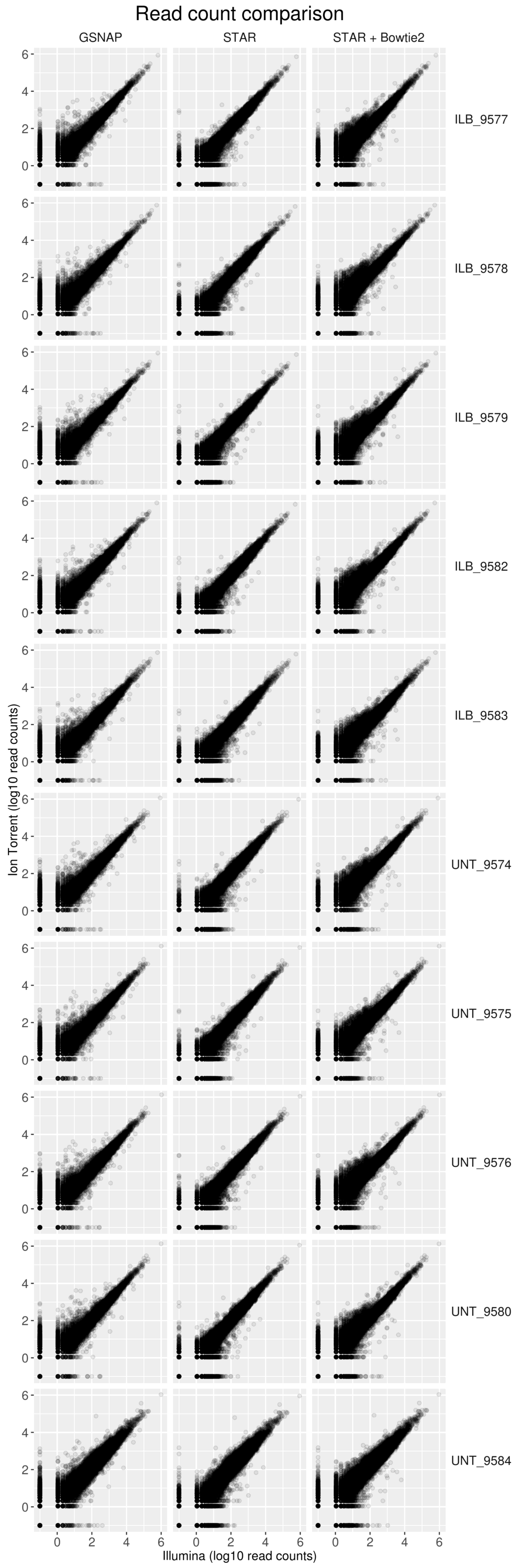
Read count comparison
GSNAP
STAR
STAR + Bowtie2
ILB_9577
ILB_9578
ILB_9579
ILB_9582
ILB_9583
UNT_9574
UNT_9575
UNT_9576
UNT_9580
UNT_9584
Ion Torrent (log10 read counts)
Illumina (log10 read counts)
0
2
4
6
