# Supplementary material for: A comparison of Illumina and Ion Torrent sequencing platforms in the context of differential gene expression
Source: BMC Genomics. 2017 Aug 10;18:602. doi: 10.1186/s12864-017-4011-0 (PMC5553782; doi:10.1186/s12864-017-4011-0)
Supplement: Supplementary file 17 — Read depth for platform-specific genes. Distributions of read counts for platform-specific genes are displayed for all samples, across all three alignment algorithms. The majority of platform-specific genes have less than 50 reads, so the graphs’ x-axes are limited to the [0, 50] range for display purposes. (PDF 1099 kb) [file 12864_2017_4011_MOESM17_ESM.pdf]

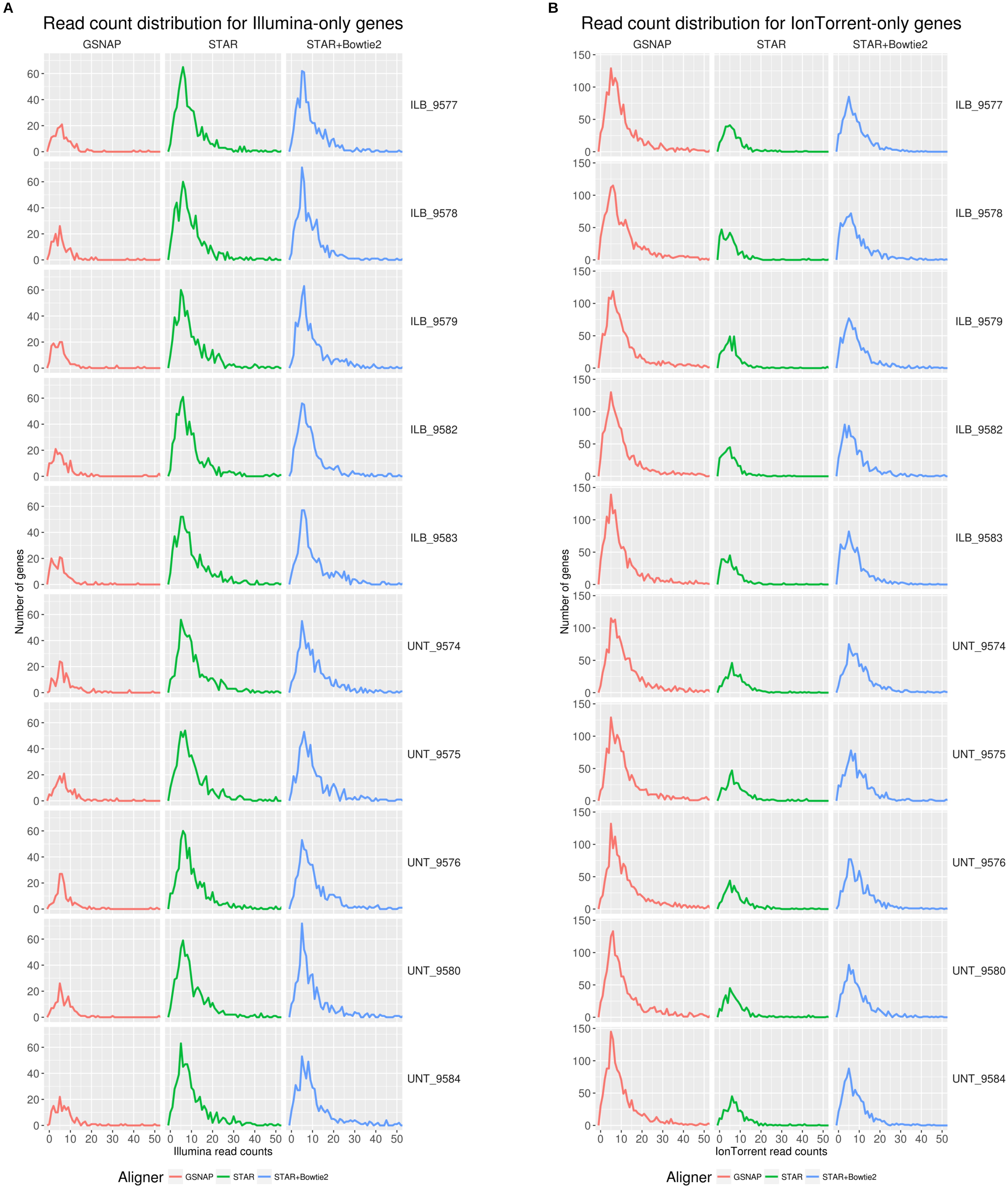
A
Read count distribution for Illumina-only genes
GSNAP
STAR
STAR+Bowtie2
Number of genes
Illumina read counts
ILB_9577
ILB_9578
ILB_9579
ILB_9582
ILB_9583
UNT_9574
UNT_9575
UNT_9576
UNT_9580
UNT_9584
Aligner
GSNAP
STAR
STAR+Bowtie2
B
Read count distribution for IonTorrent-only genes
GSNAP
STAR
STAR+Bowtie2
Number of genes
IonTorrent read counts
ILB_9577
ILB_9578
ILB_9579
ILB_9582
ILB_9583
UNT_9574
UNT_9575
UNT_9576
UNT_9580
UNT_9584
Aligner
GSNAP
STAR
STAR+Bowtie2
